# A liquid chromatography-tandem mass spectrometry based method for the quantification of adenosine nucleotides and NAD precursors and products in various biological samples

**DOI:** 10.3389/fimmu.2023.1250762

**Published:** 2023-09-20

**Authors:** Johanna Hiefner, Johann Rische, Madeleine J. Bunders, Anna Worthmann

**Affiliations:** ^1^ Department of Biochemistry and Molecular Cell Biology, University Medical Center Hamburg-Eppendorf, Hamburg, Germany; ^2^ III. Department of Medicine, University Medical Center Hamburg-Eppendorf, Hamburg, Germany; ^3^ Research Department of Virus Immunology, Leibniz Institute of Virology, Hamburg, Germany; ^4^ Hamburg Center of Translational Immunology, Hamburg, Germany

**Keywords:** metabolomics, adenine nucleotides, quantification, LC-MS/MS, HILIC, purinergic signaling

## Abstract

Adenine nucleotides (AN) are ubiquitous metabolites that regulate cellular energy metabolism and modulate cell communication and inflammation. To understand how disturbances in AN balance arise and affect cellular function, robust quantification techniques for these metabolites are crucial. However, due to their hydrophilicity, simultaneous quantification of AN across various biological samples has been challenging. Here we present a hydrophilic interaction high-performance liquid chromatography-tandem mass spectrometry (HPLC-MS/MS) based method for the quantification of 26 adenosine nucleotides and precursors as well as metabolic products of nicotinamide adenine dinucleotide (NAD) in plasma, liver, and adipose tissue samples as well as cell culture supernatants and cells. Method validation was performed with regard to linearity, accuracy, precision, matrix effects, and carryover. Finally, analysis of cell culture supernatants derived from intestinal organoids and RAW 264.7 cells illustrates that the here described method is a reliable and easy-to-use tool to quantify AN and opens up new avenues to understand the role of AN generation and breakdown for cellular functions.

## 1 Introduction

Adenine nucleotides (AN) are present in all living cells. They are crucial for metabolic processes where they serve as cofactors during energy transfer reactions. The adenosine nucleotides adenosine 5′-monophosphate (AMP), adenosine 5′- diphosphate (ADP), and adenosine 5′-triphosphate (ATP) contain one, two, or three high-energy phosphates whose hydrolysis fuels endergonic metabolic processes. Nicotinamide adenine dinucleotide (NAD) is an important cofactor for cellular redox reactions and protons derived by its reduced form nicotinamide adenine dinucleotide (NAD) + hydrogen (H), NADH, drive respiration and ATP synthesis. Together, these molecules are considered to reflect the energy status of a cell. NAD may be synthesized *de novo* from tryptophan via the kynurenine pathway or be derived from the salvage pathway via NAM and NMN (1). Besides their function in energy metabolism, AN and NAD metabolites are also involved in cellular communication. While they are known as intracellular second messengers, such as 3’,5’-cyclic adenosine monophosphate (cAMP) or the pyridine dinucleotide nicotinic acid adenine dinucleotide phosphate (NAADP), they are also found extracellularly where they modulate cell to cell communication. Of note, the release of extracellular ATP is present in plants, fungi, bacteria, insects, and mammals and controls various biological processes (2). In the context of tissue damage and inflammation, ATP release has been described as a stress or danger signal of dying cells (3) to attract phagocytes or to provide co-stimulatory signals for T-cells (4). Extracellular ATP but also ADP and AMP bind to and signal through inotropic P2XR and metabotropic P2YR receptors, which is referred to as purinergic signaling (5). In the extracellular space, ADP and AMP are derived from ATP degradation mediated by the ectonucleotidases CD39 and ENPP. AMP may also be derived from ADP-ribose resulting from CD38-mediated degradation of NAD. AMP is further converted to adenosine by CD73. Contrary to ATP, adenosine is considered to counteract inflammation and signals via the anti-inflammatory P1 receptors (6). Adenosine signaling may be terminated by cellular uptake of adenosine by equilibrative nucleoside transporters (ENTs) (7) or its degradation to inosine by adenosine deaminase (ADA).

Despite their related metabolic roles and their convertibility, the combined analysis of adenosine nucleotides, NAD metabolites, and their substrates and breakdown products is challenging. First, the main reason for this is the extreme hydrophilicity of adenosine nucleotides and in particular ATP, resulting in weak retention on reversed-phase (RP) high-performance liquid chromatography (HPLC) columns and subsequent poor separation and peak shape (8). Nevertheless, there are methods quantifying nucleotides by RP-HPLC mass spectrometry (MS) (9, 10) but these methods are limited in the amount of analytes or focus on compound identification rather than on absolute quantification. To circumvent this issue, methods using ion-pairing RP-HPLC coupled to electrospray (ESI)- MS have been introduced (11–14). Another approach is based on hydrophilic interaction liquid chromatography (HILIC). Due to their great capacity to retain polar analytes, the hydrophilic stationary phases used in HILIC offer great retention and separation of polar metabolites and HILIC has been employed for the analysis of NAD metabolites (15) but also nucleobases, nucleosides, and nucleotides (16–18). Hence, this chromatographic approach could be well suited for a method aiming to combine the quantification of these molecules. Second, extraction conditions are demanding as well. For instance, while organic extraction of NAD+ and NADH works well (19, 20), these procedures have been shown to render poor yields for breakdown products of ATP such as adenosine and inosine (20). Overall, a method for the combined analysis of adenosine nucleotides, NAD metabolites, and their substrates and breakdown products has to overcome these issues. Besides these technical challenges, most of the methods published analyze these metabolites in human plasma (10) or liver tissue (13). Given the recent advances in human 3D tissue culture models and organoid technologies, especially with regard to personalized medicine (21), analytical methods aiming to quantify intracellular and extracellular AN metabolites should be additionally validated in cells and in cell culture supernatants.

We developed a LC-MS/MS method based on HILIC-HPLC for the quantification of 26 AN and NAD metabolites which was validated in liver, adipose tissue, plasma, and cell culture supernatants and partly in cells. Applying the method to cell culture supernatants, we showed that the method detects extracellular breakdown of ATP by intestinal organoids and rapid changes in ATP release of macrophages after inflammatory stimuli.

## 2 Materials and equipment

**Table 1** gives an overview of chemicals, material, and equipment used.

**Table 1:** List of materials and equipment including vendors.

Chemicals		
Reference Standards		Vendor
Adenosine		Sigma-Aldrich
AMP		Sigma-Aldrich
ADP		Sigma-Aldrich
ATP		Sigma-Aldrich
2’desoxyATP		Sigma-Aldrich
ADPR		Sigma-Aldrich
cADPR		Sigma-Aldrich
2’desoxyADPR		Axxora
cAMP		Sigma-Aldrich
cGMP		Sigma-Aldrich
NAM		Sigma-Aldrich
NAD		Sigma-Aldrich
Reference Standards		Vendor
NADH		Sigma-Aldrich
2’NAD		Axxora
NADP		Sigma-Aldrich
NADPH		Sigma-Aldrich
NAADP		Sigma-Aldrich
NR		Sigma-Aldrich
NMN		Sigma-Aldrich
NAMN		Sigma-Aldrich
NaADN		Sigma-Aldrich
Inosine		Sigma-Aldrich
Uridine		Sigma-Aldrich
Hypoxanthine		Sigma-Aldrich
Tryptophan		Sigma-Aldrich
Kynurenine		Sigma-Aldrich
Kynurenic acid		Sigma-Aldrich
Quinolinic acid		Tocris
Hypoxanthine		Sigma-Aldrich
C13 NAM		Sigma-Aldrich
HPLC solvents		Vendor
Water, MS-grade		Supelco
Acetonitrile, MS-grade		Supelco
Ammonium acetate		Sigma-Aldrich
Eluent A		20 mM ammonium acetate in MS grade water pH 9.8
Eluent B		100% acetonitrile
Sample extraction		Vendor
Physiological Control Plasma		Sciex
Safety reaction tubes 1.5ml/2ml		Eppendorf
LPS stimulation in RAW264.7 cells		Vendor
12-well Cell culture plates		Sarstedt
RAW 264.7 macrophages		Merck
RPMI medium		Thermo Fisher
Fetal bovine serum		Thermo Fisher
Penicillin-Streptomycin		Thermo Fisher
LPS		Sigma-Aldrich
Human Intestinal Organoids		Vendor
24-well Cell culture plates		Greiner
70µM cell strainer		Falcon
Human Intestinal Organoids		Vendor
EDTA/DTT		Gibco
Basement Membrane Extract		Bio-Techne
Primocin^®^		InvivoGen
Y-27632		StemCell
DMEM/F12		Thermo Fisher
GlutaMAX		Thermo Fisher
HEPES		Thermo Fisher
penicillin/streptomycin		Sigma-Aldrich
TrypLE		Life Technologies
flat bottom 96-well plate		Sarstedt
PBS		Thermo Fisher
ATP		Sigma-Aldrich
Organoid Expansion medium (EM):		Vendor
component	final concentration	
DMEM/F12 + 1%vGlutaMAX	70% (v/v)	Thermo Fisher
B27 supplement	1x	Thermo Fisher
N2 supplement	1x	Thermo Fisher
murineEGF	50 ng/ml	Pepro-Tech
N-acetyl-L-cysteine	1.25 mM	Sigma-Aldrich
[Leu15]-Gastrin	10 nM	Sigma-Aldrich
Nicotinamide	10 nM	Sigma-Aldrich
SB202190	10 µM	Sigma-Aldrich
A83-01	500 nM	Tocris
Wnt3a Surrogate-Fc Fusion Protein	0.3 nM	ImmunoPrecise Antibodies
Noggin-CM	10% (v/v)	home-made
R-spondin-1-CM	20% (v/v)	home-made
Rho k inhibitor	10 µM	StemCell
Primocin®	100 µg/ml	InvivoGen
Devices		Vendor
General		Vendor
Centrifuges		Eppendorf, Thermofisher
Tissue Lyser		TissueLyser; QIAGEN by Retsch
Analytical Lab Balance		Sartorius
HPLC-MS/MS		Vendor
Glass vial		Phenomenex
HPLC-MS/MS		Vendor
Crimp cap		LABSOLUTE®
HPLC column Luna® 3µm NH2 100 Å 150 mm x 2 mm		Phenomenex (#00F-4377-B0)
UHPLC system Nexera X2		Shimadzu
Triple quadrupole mass spectrometer QTRAP 5500		Sciex
Analyst 1.7 software		Sciex

## 3 Methods

### 3.1 Reference standard solutions

Standard stock solutions of the pure substances were prepared individually on ice in a concentration of 1 mg/mL in MS-grade water. An 80 μM multistandard solution was prepared from the individual standards, which was diluted in acetonitrile into 13 standard levels with concentrations between 0.002 and 20 μM for calibration. In addition, an internal standard (IS) C13 NAM with a concentration of 200 μM was prepared in acetonitrile and used for quantification. The internal standard was also added to each of the 13 standard levels at a final concentration of 10 μM. The individual standards were stored at -20°C and thawed as needed. The multistandard solution and the standard levels were prepared shortly before analysis.

### 3.2 Instrumental setup

All HPLC-MS/MS analyses were performed on a triple quadrupole mass spectrometer (QTRAP 5500; SCIEX) coupled to an ultra-high pressure liquid chromatography system (Nexera X2; Shimadzu). The HPLC column Luna® 3 μm NH2 100 Å 150 mm x 2 mm was used for analyte separation. The instrument was operated with the Analyst Software (AB Sciex).

#### 3.2.1 Mass spectrometry

##### 3.2.1.1 Ion source parameters and MS parameters of analytes

The mass spectrometer was operated using Analyst software 1.7 (SCIEX) The used mass spectrometer was equipped with an ESI source. All measurements were carried out in negative ionization mode at 500°C with an ion spray voltage of -4500V. Collision gas was set to medium, ion source gas 1 and 2 were set to 40 and 20 respectively and curtain gas was set to 20. Optimized MS parameters including MRM transitions for every analyte are given in **Table 2**.

**Table 2:** MRM method with mass transitions and MS parameters for every analyte.

Q1 [Da]	Q3 [Da]	ID	DP [V]	EP [V]	CE [V]	CXP [V]	RT [min]
489.975	79.100	dATP	-125	-10	-116	-13	19.20
266.105	134.100	Adenosine	-135	-10	-28	-15	3.77
425.980	79.000	ADP	-100	-10	-80	-11	16.66
346.064	78.900	AMP	-110	-10	-70	-9	13.39
505.953	79.000	ATP	-150	-10	-96	-9	19.16
539.969	78.900	cADPR	-200	-10	-130	-9	12.17
328.048	134.000	cAMP	-105	-10	-34	-15	8.43
344.043	78.900	cGMP	-120	-10	-76	-11	10.46
742.934	620.000	NAADP	-195	-10	-26	-23	16.97
662.059	540.000	NAD	-40	-10	-22	-19	9.71
664.050	79.000	NADH	-150	-10	-128	-9	12.54
743.005	621.000	NADP	-90	-10	-26	-21	15.27
744.993	78.900	NADPH	-260	-10	-130	-11	17.75
243.070	42.000	Uridine	-40	-10	-44	-15	3.99
134.909	91.900	Hypoxanthine	-95	-10	-22	-11	6.23
266.976	134.800	Inosine	-105	-10	-28	-13	6.51
127.055	43.100	C13 NAM	-95	-10	-36	-21	2.48
120.976	42.000	NAM	-100	-10	-20	-7	2.48
646.022	524.000	dNAD	-90	-10	-22	-19	9.45
187.906	144.000	Kynurenic acid	-45	-10	-22	-17	7.75
206.912	189.900	Kynurenine	-40	-10	-12	-17	5.13
664.032	541.000	NaADN	-10	-10	-24	-21	12.11
333.936	289.900	NAMN	-35	-10	-14	-13	12.12
332.954	210.900	NMN	-35	-10	-12	-9	9.86
165.900	122.000	Quinolinic acid	-40	-10	-14	-13	13.10
202.948	116.000	Tryptophan	-70	-10	-22	-13	5.43
253.110	121.000	NR	-60	-10	-14	-13	5.04

#### 3.2.2 HPLC conditions

The HPLC system was equipped with a degasser unit, a binary pump, and a cooled autosampler. The autosampler temperature was set to 15°C for all measurements. The flow rate was set to 0.25 mL/min and the total run time was 30 min. Eluent A contained 20 mM ammonium acetate in MS-grade water (pH 9.8) and solvent B contained 100% acetonitrile. The gradient is portrayed in **Table 3**.

**Table 3:** HPLC gradient.

Time [min]	Eluent A [%]	Eluent B [%]	Flow rate [mL/min]
0.00	20	80	0.25
17.00	100	0	0.25
25.00	100	0	0.25
25.10	20	80	0.25
30.00	20	80	0.25

### 3.3 Sample extraction

For extraction, 10 μl of liquid samples such as plasma and cell culture supernatant or 5 mg of solid tissue samples such as liver and adipose tissue were transferred to ice-cooled 1.5 ml or stainless steel bead-containing 2 ml reaction tubes respectively. 10 μl of ice-cooled 200 μM C13 NAM (IS) and 180 μl (for liquid samples) or 190 μL (for solid samples) of cold acetonitrile/water ((80/20); (v/v)) were then added to the samples. For homogenization, liquid samples were vortexed at the highest level for 1 min and tissue samples were homogenized using a Tissue Lyzer for 3 min at 20 Hz. This was followed by incubation on ice for 10 min and centrifugation at 4°C and 16,000 x g for 10 min. For the measurement, the supernatant was then carefully removed with a pipette and transferred to a glass vial with an insert, which was finally tightly closed with a crimped cap. The extracts were measured immediately after extraction and then stored at -20°C.

### 3.4 Method validation

Validation of the method regarding calibration curve and range, linearity, matrix effects, accuracy and precision, and carryover, was performed according to accepted guidelines (22).

#### 3.4.1 Calibration curve, calibration range

To assess the calibration range, the 80 µM multi-Standard solution containing commercially available reference standards (prepared according to 3.1.) was diluted into 13 different calibration levels ranging from 0.002 µM to 20 µM and each level contained 10 μM IS for quantitation purposes. Calibration curves were obtained from 5 independent analytical run sequences which were measured over several days and designed by injecting the calibration levels individually in ascending order followed by blank samples to avoid carry-over after the highest calibration level. The limit of detection (LOD) was determined by the cut-off approach. For this purpose, the analyte signal area had to be 3 times higher than the analyte signal of a blank sample. The lowest limit of quantitation (LLOQ) was set as the lowest standard of calibration. Furthermore, the peak at the LLOQ had to be reproducible, identifiable, and discrete. The calibration range was described as the range between LLOQ and the upper limit of quantification (ULOQ), which was set to the highest reference standard (20 µM if not stated otherwise). Obtained analyte/internal standard peak-area ratios were plotted against concentrations (µM) and linear regression analysis was performed for two different concentration ranges. The values for LOD, LLOQ, concentration ranges, slope,intercept, and correlation coefficient are given in **Table 4**.

**Table 4:** Calibration curve parameters with limit of detection (LOD), the lower limit of qauntification (LOQ), calibration ranges, slope, and intercept (i) of the linear regression equation, and correlation coefficient (R2) Hypoxanthine; KA, Kynurenic acid; QA, Quinolinic acid.

analyte	LOD (µM)	LLOQ (µM)	Carryover (%)		Parameter linear regression							
					low range				high range			
					range (µM)	slope	i	R2	range (µM)	Slope	i	R2
dATP	<0.002	0.002	3623		0.002-0.1	0.0174	0.0001	0.9808	0.002-20	0.0201	-0.0066	0.9592
Adenosine	<0.002	0.002	29		0.002-0.1	0.9047	0.0014	0.9959	0.002-20	0.9004	-0.0239	0.9992
ADP	0.002	0.01	1451		0.01-2	0.0915	0.0042	0.9934	0.01-20	0.0935	-0.0216	0.9804
AMP	0.002	0.006	975		0.006-0.2	0.0980	0.0085	0.9770	0.006-20	0.1568	-0.0080	0.9987
ATP	<0.002	0.002	1836		0.002-0.1	0.0820	0.0013	0.9905	0.002-6	0.0416	0.0134	0.9101
cADPR	0.002	0.01	1173		0.01-0.6	0.0824	0.0004	0.9996	0.01-10	0.0.0810	-0.0079	0.9463
cAMP	0.006	0.006	0		0.006-0.2	1.0198	-0.0027	0.9882	0.006-20	0.9273	-0.0384	0.9991
cGMP	0.002	0.006	0		0.006-0.2	0.3923	0.0017	0.9987	0.006-20	0.4643	-0.0203	0.9989
NAADP	0.02	0.02	1430		0.02-1	0.0129	-0.0002	0.9988	0.02-10	0.0074	0.0023	0.9849
NAD	0.002	0.01	1308		0.01-0.6	0.1145	-0.0021	0.9953	0.01-10	0.0870	0.0026	0.9758
NADH	0.02	0.02	93		0.02-1	0.0704	-0.0023	0.9981	0.02-20	0.0505	-0.0019	0.9894
NADP	0.01	0.06	1402		0.06-2	0.0105	-0.0003	0.9995	0.06-10	0.0060	0.0024	0.9820
NADPH	0.2	0.6	0		0.6-10	0.0003	0.0006	0.9026	0.6-20	0.0005	0.0000	0.9524
Uridine	0.002	0.01	0		0.01-0.6	0.5203	0.0087	0.9929	0.01-20	0.4703	0.0078	0.9995
HX	0.06	0.06	63		0.06-2	0.7544	0.0378	0.9983	0.06-20	0.6794	0.0688	0.9993
Inosine	<0.002	0.002	0		0.002-0.1	1.4342	0.0056	0.9957	0.002-20	1.4729	0.0009	0.9997
NAM	0.06	0.06	47		0.06-2	0.1700	-0.0055	0.9975	0.06-20	0.1615	0.0027	0.9998
dNAD	0.002	0.02	607		0.02-1	0.0242	-0.0004	0.9972	0.02-20	0.0271	-0.0111	0.9708
KA	0.01	0.01	0		0.01-0.6	1.0502	-0.0182	0.9941	0.01-20	0.7399	0.0281	0.9990
Kynurenine	0.002	0.002	0		0.002-0.1	0.5446	0.0000	0.9982	0.002-20	0.5324	-0.0307	0.9983
NaADN	0.02	0.02	835		0.02-1	0.0077	-0.0001	0.9993	0.02-20	0.0081	-0.0030	0.9750
NAMN	0.06	0.1	1724		0.1-6	0.0602	0.0010	0.9984	0.1-20	0.0630	-0.0036	0.9997
NMN	0.2	0.2	0		0.2-10	0.0034	-0.0004	0.9916	0.2-20	0.0038	-0.0013	0.9954
QA	0.1	0.2	251		0.2-10	0.1598	-0.0177	0.9998	0.2-20	0.1638	-0.0278	0.9998
Tryptophan	0.01	0.01	0		0.01-0.6	0.5282	-0.0018	0.9984	0.01-20	0.4604	0.0006	0.9996
NR	0.01	0.01	0		0.01-0.6	0.2967	-0.0085	0.9921	0.01-20	0.3639	-0.0534	0.9980

#### 3.4.2 Linearity

Linearity was assessed by back-calculation of the calibrators at different concentration levels. Back-calculated concentrations together with their accuracy and precision are given in **Table 5**.

**Table 5:** Linearity given as back-calculated concentration (BCC), precision given as relative standard deviation (RSD), and accuracy given as relative error percentages (RE%) at indicated concentrations from the calibration curve (n.d. = no data).

Analyte	0.06µM			0.6µM			1µM			10µM		
	low range			low range			low range			high range		
	BCC	RSD(%)	RE(%)	BCC	RSD(%)	RE(%)	BCC	RSD(%)	RE(%)	BCC	RSD(%)	RE(%)
dATP	0.0629	12.29	4.77	0.6991	9.62	16.52	1.0740	24.77	-7.40	7.7292	11.69	22.71
Adenosine	0.0555	17.57	7.54	0.6436	10.60	7.27	1.0002	6.24	-0.02	9.7966	4.65	2.03
ADP	0.0735	23.01	22.51	0.5543	11.42	7.61	0.9011	5.40	9.89	8.8769	12.41	11.23
AMP	0.0558	38.43	6.99	0.6388	3.11	-6.47	1.0135	9.43	-1.35	9.6666	13.19	3.33
ATP	0.0562	26.16	6.34	0.6017	8.72	-0.29	0.9699	5.09	3.01	10.1891	11.89	-1.89
cADPR	0.0648	20.71	8.04	0.5171	10.12	13.82	1.1758	29.73	-17.58	9.9077	9.36	0.92
cAMP	0.0605	8.14	0.75	0.6045	10.27	7.49	1.0051	4.72	-0.51	11.0086	6.96	-10.09
cGMP	0.0648	10.79	7.95	0.7720	9.18	-28.67	1.1559	15.78	-15.59	9.9664	21.27	0.34
NAADP	0.0689	24.71	14.88	0.6944	13.22	15.73	1.0003	24.53	-0.03	10.0046	25.24	-0.05
NAD	0.5673	3.84	5.44	0.6084	6.90	1.40	0.9507	16.78	4.93	10.6782	14.55	-6.78
NADH	0.0495	19.40	17.45	0.5897	37.49	1.71	0.9926	22.85	0.74	9.2610	24.62	7.39
NADP	0.0653	27.30	8.87	0.6596	11.06	9.34	1.1406	14.99	-14.06	9.9485	25.14	0.51
NADPH	n.d.	n.d.	n.d.	0.8935	21.18	-48.92	1.8008	33.00	-80.08	8.3612	32.28	16.39
Uridine	0.0474	12.56	21.03	0.5958	14.94	0.70	0.9495	7.92	5.05	9.7914	3.23	2.09
Hypoxanthine	0.0299	50.11	83.98	0.6534	36.98	8.71	1.0668	25.55	-6.68	11.2922	21.24	-12.92
Inosine	0.0620	5.12	-3.38	0.7362	9.28	-22.70	1.1645	5.53	-16.45	11.4089	1.61	-14.09
NAM	0.0875	14.01	45.78	0.5691	5.61	5.16	1.0025	17.12	-0.25	10.2238	4.50	-2.24
dNAD	0.0489	26.31	18.45	0.6375	10.75	6.24	0.9802	15.47	1.98	8.1850	10.83	18.15
Kynurenic acid	0.0500	21.23	16.66	0.6026	12.23	0.44	0.9621	8.33	3.79	9.6103	44.78	3.90
Kynurenine	0.0571	10.11	4.82	0.5776	6.10	3.74	0.9712	7.32	2.88	9.4417	11.12	5.58
NaADN	0.0619	21.63	3.11	0.6056	12.90	0.93	1.0233	9.77	-2.33	8.3330	5.25	16.67
NAMN	n.d	n.d.	n.d.	0.6027	13.42	-0.46	1.0287	6.57	-2.87	9.8820	10.46	1.18
NMN	n.d	n.d.	n.d.	0.6611	16.77	-10.18	1.1636	17.75	-16.36	9.5914	9.44	4.09
Quinolinic acid	n.d	n.d.	n.d.	0.7270	12.27	-21.16	1.1248	3.75	-12.48	11.1299	19.31	-11.30
Tryptophan	0.0586	19.69	2.38	0.6054	6.17	-0.91	0.9179	10.84	8.21	9.7069	19.91	2.93
NR	0.0497	14.57	17.13	0.6216	11.84	3.60	1.0309	15.94	-3.09	9.6241	11.16	3.76

#### 3.4.3 Matrix effects

Matrix effects were evaluated in the matrices of liver tissue, adipose tissue, plasma, cell culture supernatant, and cells by standard addition. Matrix samples were extracted according to the protocol described in 3.3. and supernatants were pooled to create a representative matrix extract. Standard addition was performed for each concentration by adding standard solutions in 13 concentrations ranging between 0.002 μM and 20 μM. Additionally, blank samples (without standard addition and IS, n=3) and zero samples (without standard addition but with IS, n=3) were prepared and analyzed to rule out matrix effects and to determine the endogenous analyte concentration of the sample. Obtained analyte/internal standard peak-area ratios were plotted against concentrations (µM) and linear regression analysis was performed for two different concentration ranges. Values for the slope, intercept, and correlation coefficient and the back-calculated concentrations are given in **Supplementary Tables 2-6**.

#### 3.4.4 Accuracy and precision

Accuracy and precision were determined in both calibration samples as well as in all matrix-containing samples (liver, adipose tissue, plasma, cell culture supernatant, cells). Accuracy was reported as relative error percentages (RE%) of the analyte concentrations. Precision was expressed as relative standard deviation (RSD) in %. RE% and RSD were calculated using the formulas given below, where SD is the standard deviation.


$$RE\%=\frac{\left(measured\;concentration\;-\;actual\;concentration\right)}{actual\;concentration}\times 100$$

$$RSD\%=\frac{SD}{Mean}\times 100$$

All matrix samples were extracted according to the protocol described in 3.3. and the supernatants were pooled to create a representative matrix extract. The analyte concentrations to evaluate accuracy and precision were low (0.06 µM or 0.1 µM), moderate (0.6 µM, 1 µM, and 2 µM), and high (10 µM) and are given in **Table 5** or **Supplementary Tables 2-6**.

#### 3.4.5 Carryover

Carryover was assessed for each analyte during method validation by injecting numeral blank samples after the highest standard calibration level. Carryover was calculated by dividing the area of each analyte in the blank sample by the area of the same analyte in the LLOQ calibration level. The result is given as % of LLOQ.

### 3.5 Method application

#### 3.5.1 LPS stimulated ATP release in RAW264.7 cells

Raw 264.7 cells were seeded overnight at a concentration of 5 x 10^5^ cells per well in a 12-well plate in 10% FBS containing RPMI. Confluent cells were treated with LPS (10 µg/ml) or control and 20 µl supernatant was taken 5 and 10 minutes after treatment from each well. The aliquots were immediately snap-frozen in liquid nitrogen and stored on dry ice during the course of the experiment. All samples were stored at -80°C until sample extraction. Sample preparation was performed as described in 3.3. and the samples were analyzed by HPLC-MS/MS. ATP concentration was determined at 5 and 10 min after treatment for every well and ATP release/degradation was given as Δ ATP (c[ATP]_10min –_ c[ATP]_5min_) in nM.

#### 3.5.2 ATP stimulation of intestinal organoids

##### 3.5.2.1 Generation and culturing of human intestinal organoids

Human intestinal tissues were collected upon surgical procedures and intestinal tissue removal in the context of tumor resection or (re)construction of ileostomy at University Medical Centre Hamburg-Eppendorf (UKE) (Hamburg, Germany) after written informed consent of the donors (adults) or their legal guardians (children). The study to collect and analyze intestinal samples was approved by the ethics committee of the medical association of the Freie und Hansestadt Hamburg (Ärztekammer Hamburg). Human intestinal organoids were generated and cultured as described before (23–25). In short, the muscular layer and attached fat were removed and the remaining mucosal layer was cut into small pieces<0.5 cm2. The tissue was incubated for 20 minutes at 4°C on a shaker in a mixture of EDTA/DTT to isolate intestinal stem cells. Cells were filtered through a 70 µm cell strainer and centrifuged (10 minutes, 500 g, 4°C). Cells were resuspended in ice-cold Basement Membrane Extract (BME) and seeded in a pre-warmed 24-well plate. Expansion medium (EM) with 100 µg/ml Primocin^®^ and 10 µM Rho k inhibitor Y-27632 was added and changed every 2-3 days. After 10-14 days organoids were generated and subsequently passaged as follows: organoids were washed with ice-cold AD+++ (Advanced Dulbecco´s Modified Eagle´s Medium (DMEM)/F12 containing 1% GlutaMAX,10 nM HEPES and 1% penicillin/streptomycin) and disrupted mechanically through pipetting up and down. Disrupted organoids were resuspended in BME and seeded and cultured as described above.

##### 3.5.2.2 Stimulation of human intestinal organoids with ATP

To obtain a single-cell suspension, organoids were twice incubated with TrypLE for 7 minutes at 37°C while vigorously pipetting up and down multiple times. The single epithelial cells were resuspended in ice-cold BME and seeded in a flat bottom 96-well plate containing 2500 cells in on drop of 7,5 µl BME. 150 µl EM with Y-27632 per well was added and changed every 2 days. At day 4 after seeding, organoids were stimulated with PBS (control) and 10 µM ATP (Sigma-Aldrich) in EM and from there on consecutively restimulated every two days until day 12. For the here presented experiments, supernatants were collected after 48 h incubation on day 8, snap-frozen in liquid nitrogen, and kept at -80°C until further analysis. Sample extraction was performed as described in 3.3. and samples were analyzed by HPLC-MS/MS. The concentration of ATP, ADP, AMP, adenosine, inosine, and hypoxanthine was calculated and is given as Δ concentration (c_treated –_ c_PBS_) in µM.

### 3.6 Data processing and analysis

Data analysis of the MS raw data was performed using SCIEX OS software to generate quantitation reports containing peak areas. Quantification was done manually with Microsoft Excel. Linear regression of the standard calibration curve was used to quantitate accurate concentrations that reflect the analytical run performance. Prior to quantitation, all analyte areas were normalized against the area of the IS C13 NAM. Calibration levels for quantitation were adapted to low or high concentration ranges when needed. A minimum of 6 points per calibration curve was required for analyte concentration calculation.

### 3.7 Statistical analyses

Statistical analyses were conducted using GraphPad Prism 9 software. Data are shown as mean ± SEM if not indicated otherwise. Two-tailed, independent Student’s t-test was employed to compare differences between groups. Differences were considered significant at a probability level (p) of 0.05.

## 4 Results

### 4.1 HPLC-MS analysis

**Figure 1:**
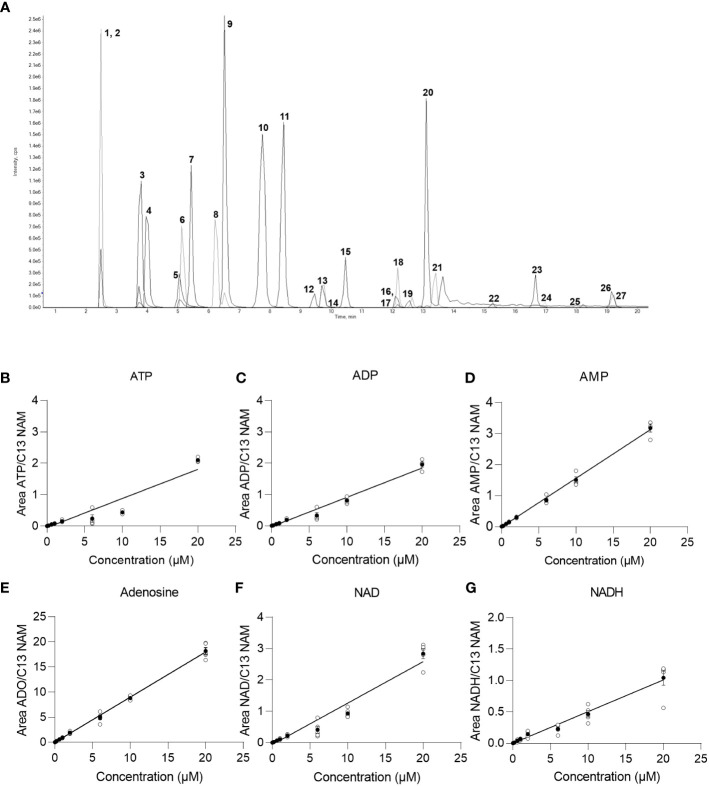
Chromatogram and baseline calibration. **(A)** Exemplary chromatogram of a 2 µM multistandard solution with 1 NAM; 2 13C NAM; 3 Adenosine; 4 Uridine; 5 NR; 6 Kynurenine; 7 Tryptophan; 8 Hypoxanthin; 9 Inosine; 10 Kynurenic acid; 11 cAMP; 12 dNAD; 13 NAD; 14 NMN; 15 cGMP; 16 NAMN; 17 NaADN; 18 cADPR; 19 NADH; 20 Quinolinic acid; 21 AMP; 22 NADP; 23 ADP; 24 NAADP; 25 NADPH; 26 ATP; 27 dATP. **(B)** linear regression of the ATP calibration curve (n=5), **(C)** linear regression of the ADP calibration curve (n=5), **(D)** linear regression of the AMP calibration curve (n=5), **(E)** linear regression of the adenosine calibration curve (n=5), **(F)** linear regression of the NAD calibration curve (n=5), **(G)** linear regression of the NADH calibration curve (n=5) with white circles show individual values and black circles show mean.

In order to detect and quantify AN and their metabolites after chromatographic separation by MS, the optimal MS parameters of the ESI source had to be established. While AN and NAD metabolites have been detected in negative, mixed (15), and positive ionization modes (9) depending on the chromatography used (13), in our hands, employing ESI in negative ionization mode rendered the most intense product ions for AN after their direct infusion into the ESI source (data not shown). Optimization of the different MS parameters was accomplished by means of the auto tuning mode provided by the Analyst 1.7. software. The optimized parameters of the multiple reaction monitoring (MRM) methods and all MS parameters are given in **Table 2**. AN have low retention on conventional RP-phases and their separation by RP-HPLC might render poor peak shapes (13). In order to increase retention and achieve better chromatographic separation and thus avoid inaccurate annotation of peaks, we employed HILIC-HPLC for the analysis of AN and NAD metabolites and breakdown products. In a first attempt, using the gradient illustrated in **Supplementary Table 1A**, clear separation of most analytes including ATP, ADP, AMP, NAD, and NADH was achieved. However, the peak shape was rather poor (**Supplementary Figure 1A**). By increasing the buffer concentration (to 20 mM) and the flow rate (to 0.25ml/min) and by using a slightly modified solvent gradient (**Table 3**), not only was total run time reduced but also a clear separation of analytes as well as improved peak shapes were achieved (**Figure 1A**; **Supplementary Figures 1B-D**).

### 4.2 Calibration curve, limit of quantification, carry over, linearity, accuracy and precision

In order to reliably quantify AN, NAD metabolites, and their building and breakdown products, external calibration and validation of the method are required. For this purpose, first, a calibration curve containing 13 calibrators ranging from 0.002 µM-20 µM was run. LOD, LLOQ, and the concentration ranges and parameters of the linear regression (slope and intercept) and carryover were determined for each analyte and are presented in **Table 4**. Representative calibration curves for ATP, ADP, AMP, Adenosine, NAD, and NADH are shown in **Figures 1B–G**. In the given concentration ranges, R^2^ was ≥0.98 for > 80% of the analytes, indicating good linear regression. Carryover varied strongly between analytes. Of note, in line with the EMA criteria (22), no or few carryover (<20% of LLOQ) was detected for cAMP, cGMP, hypoxanthine, inosine, kynurenic acid, kynurenine, NADPH, NMN, NR, quinolinic acid, and tryptophan. On the contrary, much more carryover was detected for the AN. According to their hydrophilicity, carryover gradually increased from Adenosine (29% of LLOQ) to AMP, ADP, and finally, ATP displaying a carryover of 1836% of LLOQ, indicating a very strong retention to the column.

**Figure 2:**
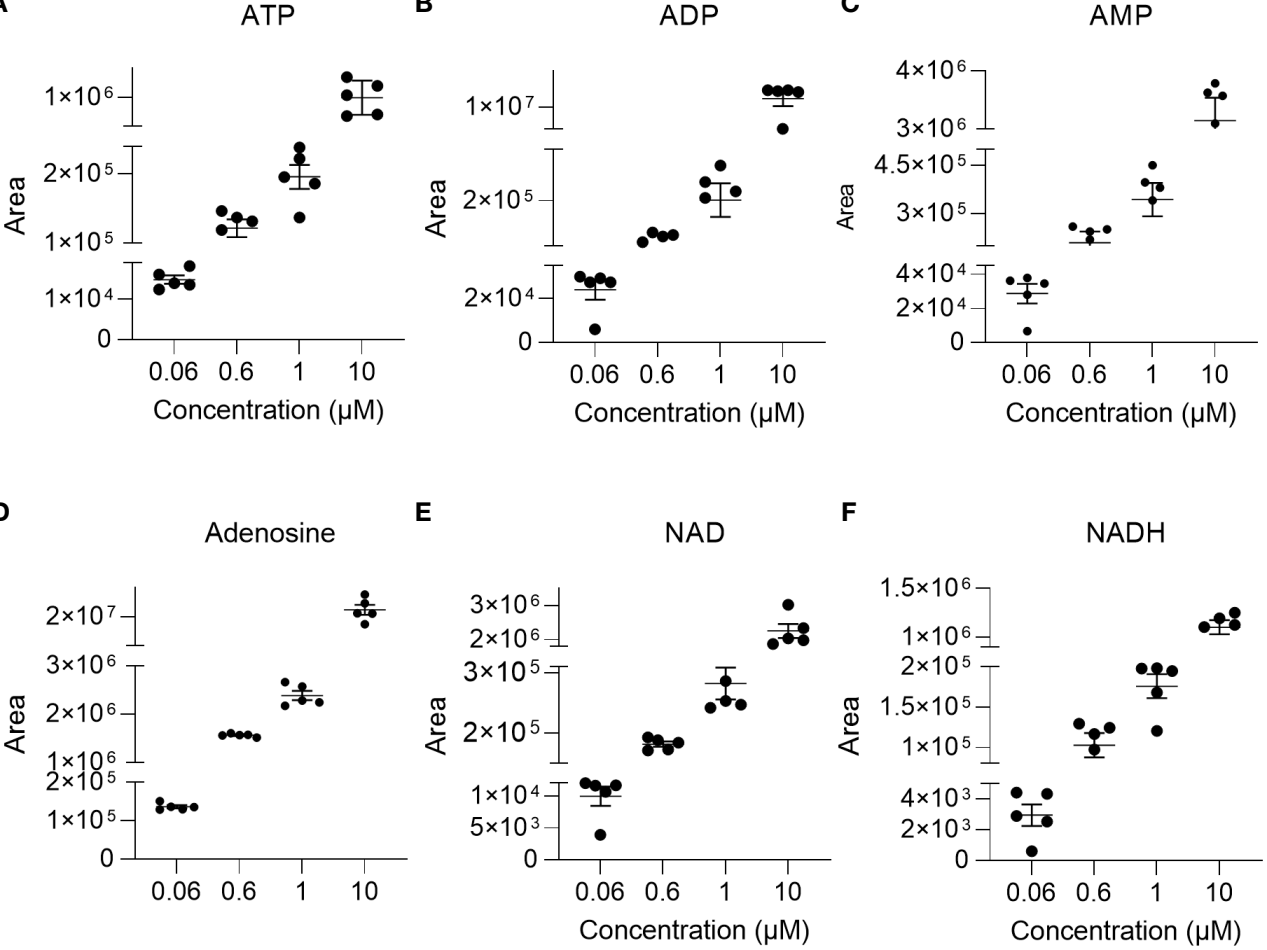
Repeatability. To illustrate repeatability, individual areas and their mean± SEM were plotted at indicated concentration levels for **(A)** ATP, **(B)** ADP, **(C)** AMP, **(D)** adenosine, **(E)** NAD, and **(F)** NADH (n=4-5).

The linearity of the method was determined by back-calculation. Back-calculated concentrations as well as accuracy and precision are presented in **Table 5**. In line with the EMA guidelines, accuracy given as RE % was below 15% for >85% of analytes at 0.06 µM and 10 µM and for roughly 80% of analytes at 0.6 µM and 1µM. Importantly, with the exception of NADH (at 0.06 µM) and ADP (at 0.06 µM), accuracy was below 15% for ATP, ADP, AMP, adenosine, NAD, and NADH at all concentrations. Additionally, as illustrated in **Figures 2A–F**, inter-day repeatability was acceptable for ATP, ADP, AMP, adenosine, NAD, and NADH. In particular, for these analytes, with the exception of NADH (at 1µM and 10 µM) and NAD (at 1µM) precision given as RSD% was below 15% at 1 µM and 10 µM and thus in line with the EMA criteria. Moreover, precision was below 20% for 70% of all analytes at 0.06 µM and below 15% for roughly >70% of all analytes at 1 µM and 10 µM, overall, indicating that the method is precise and accurate over the indicated concentration ranges.

### 4.3 Matrix effects

**Figure 3:**
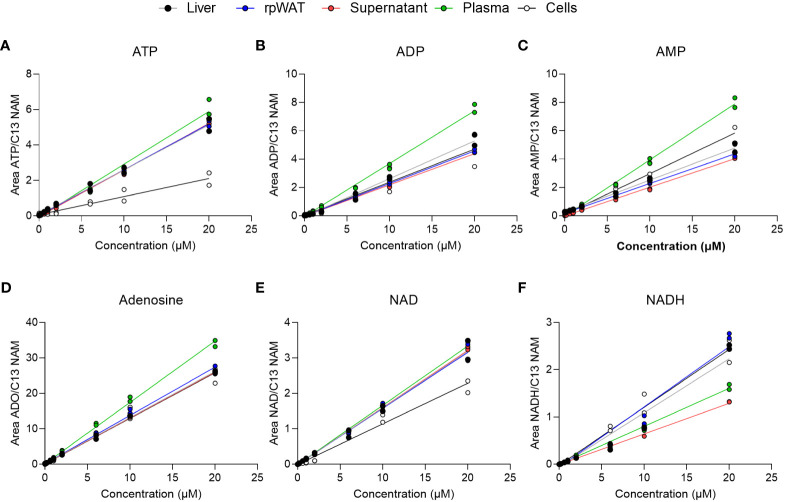
Matrix calibration. Matrix samples of liver, adipose tissue (rpWAT), supernatant, plasma, and cells were spiked with standard solutions of known concentrations. Obtained individual areas of analyte/area against the added concentration were plotted and linear regression was performed for **(A)** ATP, **(B)** ADP, **(C)** AMP, **(D)** adenosine, **(E)** NAD, and **(F)** NADH (n=2).

To evaluate matrix effects, linearity responses were studied in matrix samples after standard addition. Matrices consisted of liver, adipose tissue, plasma, cell culture supernatant, and cells. As depicted in **Figure 3**, the calibration curves of ATP, ADP, AMP, adenosine, NAD, and NADH in the matrix-containing samples were still linear. Obtained correlation coefficients of the linear regression (R^2^) were greater than 98% for 98% of analytes in supernatant, plasma, and adipose tissue, and for 96% in liver and cells (**Supplementary Tables 2-6**). Back-calculation of added concentrations was performed and back-calculated concentrations are presented in **Supplementary Tables 2-6** alongside calculated values for accuracy (as RE%) and precision (as RSD%). While in cell samples, where especially precision exceeded the EMA-tolerated 15% deviation in some analytes at 1 µM and 10 µM, accuracy and precision values obtained from the supernatant were within 15% variation for 98% of analytes at all added concentrations. Similarly, the accuracy and precision values obtained in plasma, liver, and adipose tissue samples were below 15% variation at all concentrations and thus fulfilled the EMA criteria for method validation. Moreover, inter-day repeatability was assessed in matrix-spiked samples and is given exemplary in **Supplementary Figure 2** for ATP, ADO, AMP, adenosine, NAD, and NDH, also indicating good precision.

### 4.4 Method application

In order to demonstrate the applicability of the developed method, we performed two *in-vitro* studies, where we quantified ATP as well as ATP and its degradation products ADP, AMP, adenosine, inosine, and hypoxanthine in cell culture supernatants derived from RAW264.7 cells and intestinal organoids respectively.

**Figure 4:**
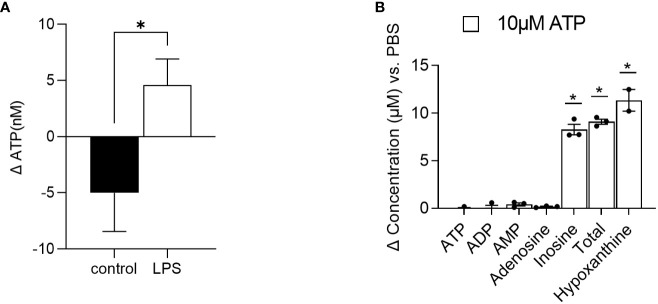
Method application. **(A)** RAW 264.7 cells were treated with LPS (n=7) or control (n=4) and ATP was quantified in the supernatant 5 and 10 minutes after stimulation. Changes in ATP levels are depicted. **(B)** Intestinal organoids were treated with 10 µM ATP and 48 h later, supernatants were collected for AN quantification. Changes in concentrations of ATP, ADP, AMP, adenosine, inosine, and hypoxanthine as well as the sum of ATP, ADP, AMP adenosine, and inosine (total) versus PBS-treated cells are depicted (n=2-3). Dates are shown as mean+ SEM. * indicates p<0.05.

First, in order to introduce an inflammatory environment, RAW264.7 cells were treated with LPS (10 µg/ml) and supernatant samples were collected 5 and 10 min after incubation. Concentrations of ATP were subsequently analyzed in the supernatants. Compared to the control-treated cells, where the concentration of ATP decreased by roughly 5 nM from 5 to 10 minutes after treatment, the concentration of ATP increased by 4.5 nM in the LPS-treated cells (**Figure 4A**), indicating ATP release. Of note, the changes in ATP levels were significantly different between the control- and LPS-treated cells.

Second, intestinal organoids were treated for 48 h with 10 µM ATP or PBS. After 48 h, concentrations of ATP and its degradation products ADP, AMP, adenosine, and inosine as well as hypoxanthine were quantified. As shown in **Figure 4B**, in the supernatants of 10 µM ATP-treated organoids, changes in ATP, ADP, AMP, and adenosine concentrations compared to the PBS-treated cells were very low (ATP: -0.124 µM, ADP: 0.019 µM, AMP: 0.405 µM, and adenosine: 0.165 µM). However, the concentration of the final ATP degradation products inosine and further hypoxanthine increased by 8.28 µM and 11.34 µM respectively, indicating that ATP was rapidly degraded into inosine and hypoxanthine.

## 5 Discussion

AN are central metabolites for the regulation of energy metabolism and are involved in cell-to-cell communication as well as intracellular signaling. During inflammatory processes, their rapid generation and breakdown critically affect their function. To understand these processes, an accurate and reliable quantification of AN is required. Previously, luciferase-based methods enabled the rapid, sensitive, and reliable determination of ATP levels but were restricted to one metabolite only (26, 27). While gas chromatography-MS/MS-based techniques have been published for the analysis of the kynurenine pathway and provided great sensitivity, sample preparation involved laborious derivatization (28). Further, ATP and its breakdown products have been quantified using HPLC and fluorescence detection (29). Here, the quantification relies on the retention time only, and thus, a drawback of this approach is the potential interference between metabolites due to undesired sample degradation. This can be overcome by the application of MS/MS methods and indeed a number of LC-MS/MS-based approaches for the analysis of AN or NAD metabolites and their breakdown products exist. However, these methods are either limited in the coverage of analytes (9), rely on complicated extraction procedures using solid phase extraction (9), or are restricted with regard to the validated matrix (13, 14). Here, we present a LC-MS/MS-based method for the accurate quantification of adenosine nucleotides and NAD metabolites (either synthesized from tryptophan via the kynurenine pathway or generated via the salvage pathway) in various biological samples. Our method stands out for the broad spectrum of metabolites, the fast and simple sample cleanup, specific mass transitions for each compound, and a great number of validated matrices. In order to meet the chromatographic challenge of not only the number of analytes but also their hydrophilicity, an amino phase and HILIC-HPLC was employed as described by Bustamante et al. (15). To further improve chromatographic separation and peak shape, buffer concentrations were increased to 20 mM which caused higher polarity of the buffer and therefore lowered retention times of the polar analytes such as ATP. This relationship of buffer concentration and nucleotide retention on the stationary phase in a HILIC approach was already described by Padivitage et al. (30). In addition, using HILIC has also other advantages compared to other chromatography approaches. First, HILIC columns require reduced column maintenance compared to porous graphitic carbon stationary phases which have been previously used for nucleotide analysis (31). Further, by employing HILIC, one can avoid the use of ion-pair reagents such as dibutylammonium acetate (13).

Using the here described optimized LC approach, our method was validated according to the accepted EMA guidelines (22). Over the indicated concentration ranges, the linearity, accuracy, and precision varied less than 15% for more than 80% of the studied analytes. In addition, the accuracy and precision fulfilled the EMA criteria for the matrices of liver, adipose tissue, plasma, and cell culture supernatant, indicating a valid and reliable method. For cells, the method is only partly validated, as EMA criteria were met for some analytes. Generally, our method might be expanded in order to investigate other matrices such as urine or cerebrospinal fluid, which have already been studied by other published methods (32, 33). Of note, these matrices should be validated and especially, their suitability regarding analyte stability needs to be assessed. For instance, in saliva, possible challenges might involve the rapid degradation of ATP and other AN by ectonucleotidases (34). Nevertheless, to our knowledge, validation for this many different matrices is a unique feature of our method and thus advantageous compared to existing methods. Furthermore, the high number of matrices suggests the great applicability of the method across various tissues and for a broad spectrum of scientific questions. As required by the EMA, the carryover rates were assessed but yielded partly disappointing results. The very hydrophilic compounds, ATP and ADP, especially showed extremely high carryover. Of note, in line with the EMA guideline (22), when carryover seems unavoidable, as in our case for AN, one should keep in mind to not randomize study samples upon designing analysis batches and to build measures in order to prevent carryover to study samples. This can be achieved by the injection of several blank samples after samples and calibrators with expected high concentrations or by rinsing approaches. Ultimately, we demonstrated that the method can be applied across tissues for various biologically relevant questions.

Besides the evaluation of ATP levels in plasma or liver tissue to evaluate energy status (13), the quantification of extracellular ATP and its degradation products may be of the utmost interest when studying inflammatory processes, as ATP is released upon stress responses (6). By applying our method, we show that ATP is rapidly released from RAW 264.7 cells upon LPS stimulation. Using a luciferase assay, Sakaki et al. (35) described a similar increase in ATP levels in LPS-stimulated THP-1 cells. Moreover, we also showed that intestinal organoids are able to break down exogenous ATP in the supernatant within 48 h into mainly inosine and further hypoxanthine. Of note, the added exogenous amount of 10 µM was roughly recovered as the sum of all degradation products (ATP, ADP, AMP, adenosine, and inosine =total) with a concentration of 9.1 µM and the final product hypoxanthine with a concentration of 11.34 µM, underlining the reliability of the method.

Although we provided examples of the applicability of the method for real samples, users might consider the potential limitations of our method. First, even though we did a thorough validation across various tissues, endogenous concentrations for some of the metabolites are really low in some tissues (e.g. concentration of NAM or kynurenic acid are at the low nanomolar ranges in plasma based on HMDB), and thus at the lower limits of quantification. Additionally, for different cell types and adipose tissues, the endogenous concentration of metabolites is currently unknown and may therefore be below our quantification limit. Hence, other techniques with higher sensitivity e.g. GC-MS might be more suitable for their analysis. For instance, while the LOD of quinolinic acid was 100 nM in our method, a GC-based analysis was able to detect 1 nM (28). Theoretically, low endogenous levels of analytes might be increased by simply using more material for extraction and concentration of the analytes. However, as the stability of AN is a huge issue, sample preparation that includes sample concentration might result in unwanted degradation and inaccurate results. Of note, due to the poor stability of analytes, care should be taken regarding sample handling (e.g. immediate quenching and/or cooling) in order to prevent degradation. As already discussed, another problem is the hydrophilicity of the analytes. Their strong retention to the column causes significant carryover which has to be considered when designing sample batches.

Altogether, we developed and validated a HILIC-LC-MS/MS-based method for the quantification of adenine nucleotides as well as NAD metabolites in liver, adipose tissue, plasma, cell culture supernatants, and cells. We demonstrated that our method can detect rapid changes in the nM to µM range in ATP as well as its degradation products in sophisticated *in vitro* models. Our method offers a powerful tool to quantify AN across various biological samples and may help to improve the understanding of AN generation and breakdown for cellular functions.

## Data availability statement

The original contributions presented in the study are included in the article/**Supplementary Material**. Further inquiries can be directed to the corresponding author.

## Ethics statement

The studies involving humans were approved by Ethics committee of the medical association of the Freie und Hansestadt Hamburg (Ärztekammer 189 Hamburg). The studies were conducted in accordance with the local legislation and institutional requirements. The participants provided their written informed consent to participate in this study.

## Author contributions

JH generated, analyzed, and visualized data and edited the manuscript. JR generated organoids data and was supervised by MJB. AW conceptualized and supervised the study, analyzed and visualized data, and wrote the manuscript. All authors contributed to the article and approved the submitted version.

## References

[1] Covarrubias AJ Perrone R Grozio A Verdin E . Nad(+) metabolism and its roles in cellular processes during ageing. Nat Rev Mol Cell Biol (2021) 22(2):119–41. doi: 10.1038/s41580-020-00313-x PMC7963035 33353981

[2] Verkhratsky A Burnstock G . Biology of purinergic signalling: its ancient evolutionary roots, its omnipresence and its multiple functional significance. Bioessays (2014) 36(7):697–705. doi: 10.1002/bies.201400024 24782352

[3] Chekeni FB Elliott MR Sandilos JK Walk SF Kinchen JM Lazarowski ER . Pannexin 1 channels mediate 'Find-me' Signal release and membrane permeability during apoptosis. Nature (2010) 467(7317):863–7. doi: 10.1038/nature09413 PMC3006164 20944749

[4] Atarashi K Nishimura J Shima T Umesaki Y Yamamoto M Onoue M . Atp drives lamina propria T(H)17 cell differentiation. Nature (2008) 455(7214):808–12. doi: 10.1038/nature07240 18716618

[5] Burnstock G . Purine and purinergic receptors. Brain Neurosci Adv (2018) 2:23982128188174945. doi: 10.1177/2398212818817494 PMC7058212 32166165

[6] Cekic C Linden J . Purinergic regulation of the immune system. Nat Rev Immunol (2016) 16(3):177–92. doi: 10.1038/nri.2016.4 26922909

[7] Baldwin SA Beal PR Yao SY King AE Cass CE Young JD . The equilibrative nucleoside transporter family, slc29. Pflugers Arch (2004) 447(5):735–43. doi: 10.1007/s00424-003-1103-2 12838422

[8] Qin X Wang X . Quantification of nucleotides and their sugar conjugates in biological samples: purposes, instruments and applications. J Pharm BioMed Anal (2018) 158:280–875. doi: 10.1016/j.jpba.2018.06.013 29902692

[9] Jimmerson LC Bushman LR Ray ML Anderson PL Kiser JJ . A lc-ms/ms method for quantifying adenosine, guanosine and inosine nucleotides in human cells. Pharm Res (2017) 34(1):73–83. doi: 10.1007/s11095-016-2040-z 27633886 PMC5177511

[10] He L Wei X Ma X Yin X Song M Donninger H . Simultaneous quantification of nucleosides and nucleotides from biological samples. J Am Soc Mass Spectrom (2019) 30(6):987–1000. doi: 10.1007/s13361-019-02140-7 30847833 PMC6520184

[11] Knee JM Rzezniczak TZ Barsch A Guo KZ Merritt TJ . A novel ion pairing lc/ms metabolomics protocol for study of a variety of biologically relevant polar metabolites. J Chromatogr B Analyt Technol BioMed Life Sci (2013) 936:63–735. doi: 10.1016/j.jchromb.2013.07.027 24004912

[12] Wu J Zhang Y Wiegand R Wang J Bepler G Li J . Quantitative analysis of intracellular nucleoside triphosphates and other polar metabolites using ion pair reversed-phase liquid chromatography coupled with tandem mass spectrometry. J Chromatogr B Analyt Technol BioMed Life Sci (2015) 1006:167–78. doi: 10.1016/j.jchromb.2015.10.030 PMC6130891 26551209

[13] Fu X Deja S Kucejova B Duarte JAG McDonald JG Burgess SC . Targeted determination of tissue energy status by lc-ms/ms. Anal Chem (2019) 91(9):5881–87. doi: 10.1021/acs.analchem.9b00217 PMC6506803 30938977

[14] Gill BD Indyk HE Manley-Harris M . Analysis of nucleosides and nucleotides in infant formula by liquid chromatography-tandem mass spectrometry. Anal Bioanal Chem (2013) 405(15):5311–9. doi: 10.1007/s00216-013-6935-9 23559337

[15] Bustamante S Jayasena T Richani D Gilchrist RB Wu LE Sinclair DA . Quantifying the cellular nad+ Metabolome using a tandem liquid chromatography mass spectrometry approach. Metabolomics (2017) 14(1):15. doi: 10.1007/s11306-017-1310-z 30830318 PMC6519110

[16] Sakaguchi Y Miyauchi K Kang BI Suzuki T . Nucleoside analysis by hydrophilic interaction liquid chromatography coupled with mass spectrometry. Methods Enzymol (2015) 560:19–285. doi: 10.1016/bs.mie.2015.03.015 26253964

[17] Tang DQ Zou L Yin XX Ong CN . Hilic-ms for metabolomics: an attractive and complementary approach to rplc-ms. Mass Spectrom Rev (2016) 35(5):574–600. doi: 10.1002/mas.21445 25284160

[18] Guo S Duan JA Qian D Wang H Tang Y Qian Y . Hydrophilic interaction ultra-high performance liquid chromatography coupled with triple quadrupole mass spectrometry for determination of nucleotides, nucleosides and nucleobases in ziziphus plants. J Chromatogr A (2013) 1301:147–555. doi: 10.1016/j.chroma.2013.05.074 23800804

[19] Sporty JL Kabir MM Turteltaub KW Ognibene T Lin SJ Bench G . Single sample extraction protocol for the quantification of nad and nadh redox states in saccharomyces cerevisiae. J Sep Sci (2008) 31(18):3202–11. doi: 10.1002/jssc.200800238 PMC2640230 18763242

[20] Noack H Kunz WS Augustin W . Evaluation of a procedure for the simultaneous determination of oxidized and reduced pyridine nucleotides and adenylates in organic phenol extracts from mitochondria. Anal Biochem (1992) 202(1):162–5. doi: 10.1016/0003-2697(92)90222-S 1621979

[21] Bose S Clevers H Shen X . Promises and challenges of organoid-guided precision medicine. Med (2021) 2(9):1011–26. doi: 10.1016/j.medj.2021.08.005 PMC8492003 34617071

[22] EMA . Ich M10 on Bioanalytical Method Validation - Scientific Guideline, in: European Medicines Agency (Accessed 26.09.2022).

[23] Schreurs R Baumdick ME Sagebiel AF Kaufmann M Mokry M Klarenbeek PL . Human fetal tnf-alpha-cytokine-producing cd4(+) effector memory T cells promote intestinal development and mediate inflammation early in life. Immunity (2019) 50(2):462–76.e8. doi: 10.1016/j.immuni.2018.12.010 30770246

[24] Sato T Stange DE Ferrante M Vries RG Van Es JH Van den Brink S . Long-term expansion of epithelial organoids from human colon, adenoma, adenocarcinoma, and barrett's epithelium. Gastroenterology (2011) 141(5):1762–72. doi: 10.1053/j.gastro.2011.07.050 21889923

[25] Sato T Vries RG Snippert HJ van de Wetering M Barker N Stange DE . Single lgr5 stem cells build crypt-villus structures in vitro without a mesenchymal niche. Nature (2009) 459(7244):262–5. doi: 10.1038/nature07935 19329995

[26] Prioli RP Tanna A Brown IN . Rapid methods for counting mycobacteria–comparison of methods for extraction of mycobacterial adenosine triphosphate (Atp) determined by firefly luciferase assay. Tubercle (1985) 66(2):99–108. doi: 10.1016/0041-3879(85)90074-1 3895682

[27] Askgaard DS Gottschau A Knudsen K Bennedsen J . Firefly luciferase assay of adenosine triphosphate as a tool of quantitation of the viability of bcg vaccines. Biologicals (1995) 23:55–60. doi: 10.1016/1045-1056(95)90012-8 7619437

[28] Notarangelo FM Wu HQ Macherone A Graham DR Schwarcz R . Gas chromatography/tandem mass spectrometry detection of extracellular kynurenine and related metabolites in normal and lesioned rat brain. Anal Biochem (2012) 421(2):573–81. doi: 10.1016/j.ab.2011.12.032 PMC3274646 22239963

[29] Ledderose C Valsami EA Junger WG . Optimized hplc method to elucidate the complex purinergic signaling dynamics that regulate atp, adp, amp, and adenosine levels in human blood. Purinergic Signal (2022) 18(2):223–39. doi: 10.1007/s11302-022-09842-w PMC9123122 35132577

[30] Padivitage NL Dissanayake MK Armstrong DW . Separation of nucleotides by hydrophilic interaction chromatography using the frulic-N column. Anal Bioanal Chem (2013) 405(27):8837–48. doi: 10.1007/s00216-013-7315-1 23995506

[31] Trammell SA Brenner C . Targeted, lcms-based metabolomics for quantitative measurement of nad(+) metabolites. Comput Struct Biotechnol J (2013) 4:e2013010125. doi: 10.5936/csbj.201301012 PMC3962138 24688693

[32] Cubbon S Bradbury T Wilson J Thomas-Oates J . Hydrophilic interaction chromatography for mass spectrometric metabonomic studies of urine. Anal Chem (2007) 79(23):8911–8. doi: 10.1021/ac071008v 17973349

[33] Schwieler L Trepci A Krzyzanowski S Hermansson S Granqvist M Piehl F . A novel, robust method for quantification of multiple kynurenine pathway metabolites in the cerebrospinal fluid. Bioanalysis (2020) 12(6):379–92. doi: 10.4155/bio-2019-0303 PMC9472175 32209024

[34] Gonzalez DA Barbieri van Haaster MM Quinteros Villarruel E Hattab C Ostuni MA Orman B . Salivary extracellular vesicles can modulate purinergic signalling in oral tissues by combined ectonucleoside triphosphate diphosphohydrolases and ecto-5'-nucleotidase activities. Mol Cell Biochem (2020) 463(1-2):1–11. doi: 10.1007/s11010-019-03624-6 31531757

[35] Sakaki H Tsukimoto M Harada H Moriyama Y Kojima S . Autocrine regulation of macrophage activation via exocytosis of atp and activation of P2y11 receptor. PloS One (2013) 8(4):e59778. doi: 10.1371/journal.pone.0059778 23577075 PMC3618444

