# Supplementary material for: A liquid chromatography-tandem mass spectrometry based method for the quantification of adenosine nucleotides and NAD precursors and products in various biological samples
Source: Front Immunol. 2023 Sep 20;14:1250762. doi: 10.3389/fimmu.2023.1250762 (PMC10548204; doi:10.3389/fimmu.2023.1250762)
Supplement: Supplementary file 1 [file DataSheet_1.docx]

Supplementary Material

# Supplementary Figures


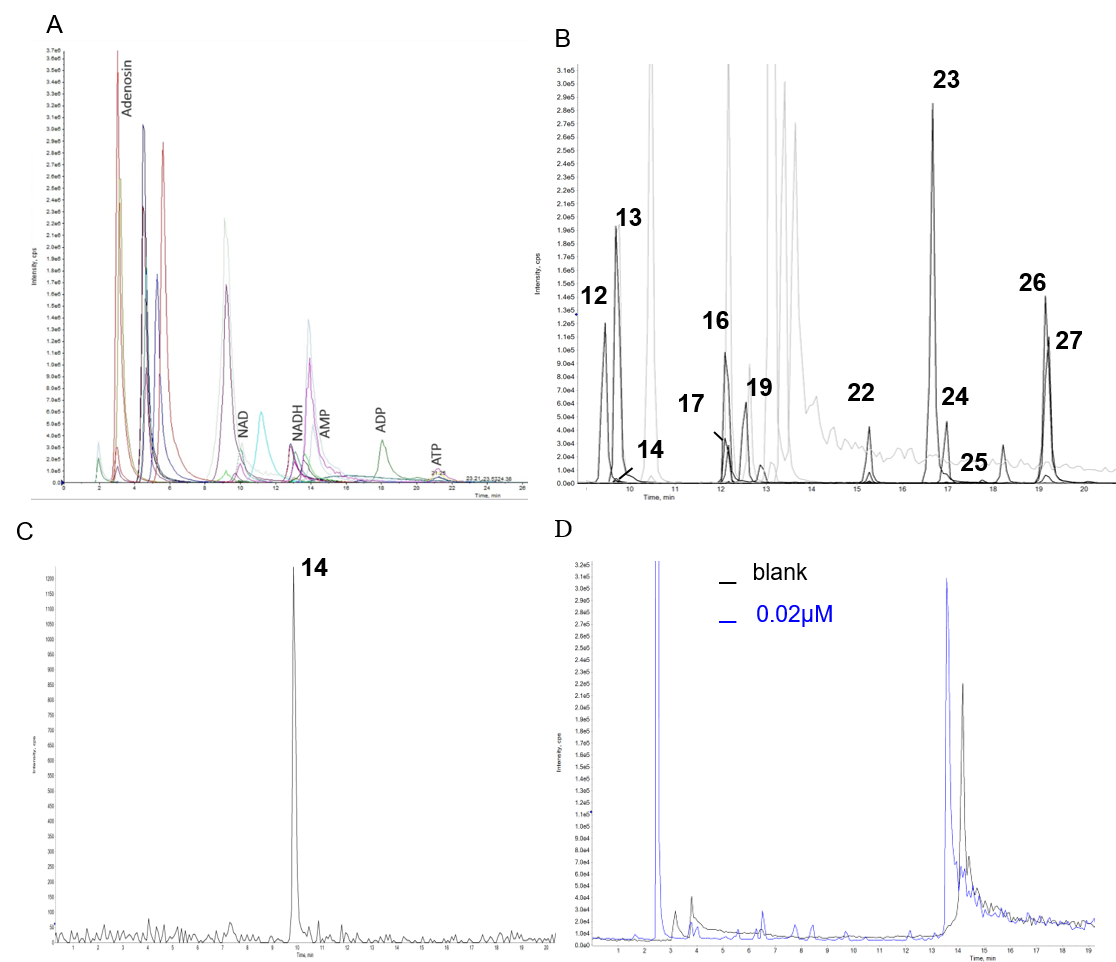


**Supplementary Figure 1.** (A) Exemplary chromatogram of a 10 µM multistandard solution using a first attempt gradient shown in Suppl. Table 1. (B) Magnification of the chromatogram given in Figure 1A (2 µM multistandard solution) to illustrate less intense compounds with: **12** dNAD; **13** NAD; **14** NMN; **16** NAMN; **17** NaADN; **19** NADH; **22** NADP; **23** ADP; **24** NAADP; **25** NADPH; **26** ATP; **27** dATP. (C) XIC for **14** NMN. (D) Chromatogram (TIC) of blank sample (black line) and multistandard solution (0.6µM) (blue line).

**
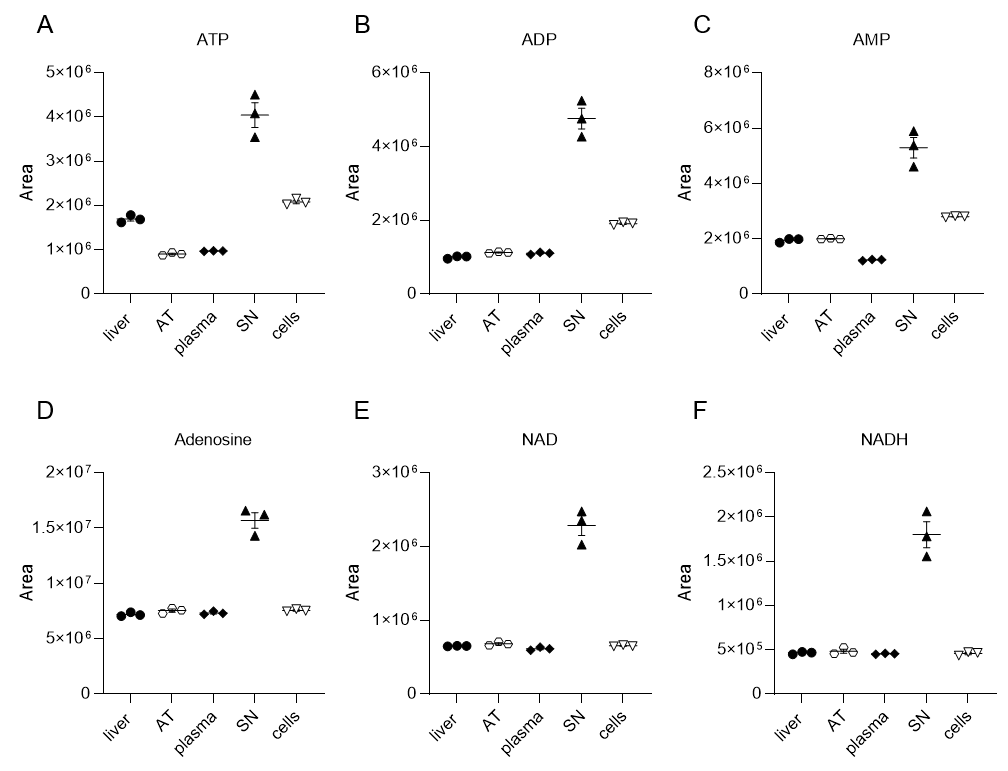
**

**Supplementary Figure 2.** Matrix Repeatability

To illustrate repeatability, individual areas as well as their mean± SEM were plotted at indicated concentration levels in indicated matrices for (A) ATP, (B) ADP, (C) AMP, (D) adenosine, (E) NAD, and (F) NADH.

# Supplementary Tables

**Supplementary Table 1: HPLC gradient and flow rate of the first HILIC attempt**

| Time [min] | Solvent A [%] | Solvent B [%] | Flow rate [mL/min] |
| --- | --- | --- | --- |
| 0.00 | 20 | 80 | 0.2 |
| 20.00 | 100 | 0 | 0.2 |
| 30.00 | 100 | 0 | 0.2 |
| 30.10 | 20 | 80 | 0.2 |
| 48.00 | 20 | 80 | 0.2 |

**Suppl.Table 2:** V**alidation in supernatant slope and intercept (i) of linear regression equation, and correlation coefficient (R2) as well as linearity given as back-calculated concentration (BCC), accuracy as relative error percentages (RE%) and as relative standard deviation (RSD) at given added concentrations.**

| Analyte | Parameter linear regression | | | | | | Added concentration | | | | | | | | | | | | |
| --- | --- | --- | --- | --- | --- | --- | --- | --- | --- | --- | --- | --- | --- | --- | --- | --- | --- | --- | --- |
|  | range(µM) 0.01-0.6 | | | range(µM) 0.02-20 | | | 0.1µM (range 0.01-0.6) | | | 1µM (range 0.02-20) | | | 2µM (range 0.02-20) | | | 10µM (range 0.02-20) | | | |
|  | slope | i | R2 | slope | i | R2 | BCC | RE(%) | RSD(%) | BCC | RE(%) | RSD(%) | BCC | RE(%) | RSD(%) | BCC | RE(%) | RSD(%) |  |
| dATP | 0.0640 | 0.0012 | 0.9993 | 0.0799 | -0.0116 | 0.9983 | 0.0967 | -3.26 | 12.17 | 0.9739 | -2.61 | 5.62 | 1.9032 | -4.84 | 1.83 | 9.4500 | -5.50 | 5.94 |  |
| Adenosine | 1.4699 | 0.0035 | 0.9999 | 1.2856 | 0.2211 | 0.9980 | 0.1038 | 3.80 | 2.12 | 0.9589 | -4.11 | 2.12 | 2.1387 | 6.93 | 2.19 | 10.3344 | 3.34 | 2.46 |  |
| ADP | 0.1804 | 0.0093 | 0.9998 | 0.2209 | -0.0248 | 0.9982 | 0.1025 | 2.48 | 3.60 | 0.9545 | -4.55 | 1.42 | 1.9177 | -4.12 | 1.20 | 9.3735 | -6.27 | 1.93 |  |
| AMP | 0.2063 | 0.0079 | 0.9998 | 0.2023 | -0.0124 | 0.9978 | 0.1053 | 5.28 | 0.79 | 1.0436 | 4.36 | 2.26 | 2.0085 | 0.43 | 1.32 | 9.2927 | -7.07 | 2.99 |  |
| ATP | 0.1921 | 0.0014 | 0.9984 | 0.2639 | -0.0502 | 0.9978 | 0.0955 | -4.45 | 24.89 | 0.9917 | -0.83 | 2.69 | 1.8441 | -7.79 | 0.28 | 9.3631 | -6.37 | 4.65 |  |
| cADPR | 0.2146 | -0.0005 | 0.9996 | 0.2268 | -0.0253 | 0.9981 | 0.1030 | 2.97 | 7.48 | 1.0050 | 0.50 | 2.25 | 1.9623 | -1.88 | 2.73 | 9.2991 | -7.01 | 1.28 |  |
| cAMP | 0.6523 | 0.0004 | 0.9998 | 0.6918 | -0.0615 | 0.9990 | 0.1012 | 1.20 | 6.03 | 1.0170 | 1.70 | 0.21 | 1.9410 | -2.95 | 0.95 | 9.4952 | -5.05 | 0.67 |  |
| cGMP | 0.3144 | -0.0001 | 0.9999 | 0.3281 | -0.0274 | 0.9991 | 0.1034 | 3.44 | 3.11 | 1.0278 | 2.78 | 1.51 | 1.9814 | -0.93 | 0.29 | 9.5984 | -4.02 | 4.83 |  |
| NAADP | 0.0271 | -0.0004 | 0.9959 | 0.0402 | -0.0098 | 0.9959 | 0.0886 | -11.39 | 10.27 | 0.9884 | -1.16 | 1.47 | 1.9242 | -3.79 | 4.39 | 9.0674 | -9.33 | 1.63 |  |
| NAD | 0.1468 | -0.0009 | 0.9991 | 0.1618 | -0.0193 | 0.9984 | 0.1055 | 5.53 | 3.69 | 1.0249 | 2.49 | 1.99 | 1.9601 | -2.00 | 5.75 | 9.3717 | -6.28 | 0.08 |  |
| NADH | 0.0725 | -0.0012 | 0.9968 | 0.0649 | -0.0066 | 0.9972 | 0.0791 | -20.89 | 7.06 | 1.0413 | 4.13 | 2.57 | 2.1478 | 7.39 | 2.09 | 9.2531 | -7.47 | 0.09 |  |
| NADP | 0.0212 | -0.0004 | 0.9928 | 0.0453 | -0.0120 | 0.9981 | 0.0986 | -1.44 | 19.50 | 0.9118 | -8.82 | 2.24 | 1.7855 | -10.72 | 0.75 | 9.4885 | -5.12 | 2.10 |  |
| NADPH | n.d. | n.d. | n.d. | 0.0031 | 0.0000 | 0.9994 | n.d. | n.d. | n.d. | 1.0561 | 5.61 | 18.58 | 1.9994 | -0.03 | 1.05 | 9.5940 | -4.06 | 4.27 |  |
| Uridine | 0.6745 | 0.0091 | 0.9997 | 0.5582 | 0.0914 | 0.9990 | 0.1057 | 5.66 | 4.17 | 0.9972 | -0.28 | 1.19 | 2.1463 | 7.31 | 0.54 | 10.1191 | 1.19 | 1.03 |  |
| Hypoxanthine | 0.6074 | 0.0195 | 0.9999 | 0.6002 | 0.0238 | 0.9999 | 0.1039 | 3.93 | 3.00 | 0.9861 | -1.39 | 0.33 | 2.0082 | 0.41 | 3.14 | 9.8622 | -1.38 | 1.50 |  |
| Inosine | 1.3069 | 0.0074 | 0.9999 | 1.2159 | 0.1425 | 0.9989 | 0.1024 | 2.42 | 1.90 | 0.9485 | -5.15 | 0.45 | 2.0759 | 3.80 | 0.57 | 10.2166 | 2.17 | 0.30 |  |
| NAM | 0.1975 | 0.0854 | 0.9959 | 0.1493 | 0.1179 | 0.9980 | 0.1196 | 19.57 | 15.59 | 1.0017 | 0.17 | 6.60 | 2.2067 | 10.33 | 6.24 | 10.3304 | 3.30 | 0.55 |  |
| dNAD | 0.0438 | 0.0000 | 0.9997 | 0.0454 | -0.0042 | 0.9989 | 0.0952 | -4.83 | 0.31 | 1.0126 | 1.26 | 3.03 | 1.9670 | -1.65 | 3.12 | 9.5180 | -4.82 | 1.31 |  |
| Kynurenic acid | 0.3803 | 0.0025 | 0.9995 | 0.8294 | -0.0346 | 0.9847 | 0.1053 | 5.30 | 1.84 | 0.5074 | -49.26 | 5.85 | 0.9862 | -50.69 | 0.05 | 11.0483 | 10.48 | 20.26 |  |
| Kynurenine | 0.6874 | 0.0392 | 0.9997 | 0.6830 | 0.0573 | 0.9999 | 0.1083 | 8.28 | 9.37 | 0.9673 | -3.27 | 0.75 | 2.0323 | 1.62 | 3.70 | 9.9677 | -0.32 | 1.32 |  |
| NaADN | 0.0189 | -0.0001 | 0.9999 | 0.0189 | -0.0014 | 0.9987 | 0.1016 | 1.65 | 15.34 | 1.0403 | 4.03 | 0.83 | 1.9975 | -0.12 | 0.31 | 9.4256 | -5.74 | 2.59 |  |
| NAMN | 0.0732 | 0.0005 | 0.9999 | 0.0814 | -0.0094 | 0.9984 | 0.1015 | 1.50 | 15.23 | 1.0352 | 3.52 | 3.02 | 1.9622 | -1.89 | 0.87 | 9.4265 | -5.73 | 5.04 |  |
| NMN* | 0.0036 | 0.0000 | 0.9983 | 0.0040 | -0.0005 | 0.9986 | 0.11422049 | 14.22 | 2.04 | 1.0159 | 1.59 | 1.65 | 2.0137 | 0.68 | 2.33 | 9.4519 | -5.48 | 0.41 |  |
| Quinolinic acid# | n.d. | n.d. | n.d. | 0.390185 | -0.114663 | 0.994323 | n.d. | n.d. | n.d. | 1.0890 | 8.90 | 2.22 | 1.9641 | -1.79 | 3.57 | 8.9383 | -10.62 | 1.41 |  |
| Tryptophan | 0.4496 | 1.5643 | 0.8806 | 0.5537 | 1.5445 | 0.9998 | 0.2659 | 165.95 | 91.29 | 0.9619 | -3.81 | 4.18 | 1.9298 | -3.51 | 6.53 | 9.8960 | -1.04 | 0.93 |  |
| NR | 0.3396 | 0.0024 | 0.9999 | 0.4357 | -0.0840 | 0.9980 | 0.1039 | 3.93 | 4.77 | 0.9568 | -4.32 | 7.48 | 1.7920 | -10.40 | 3.60 | 9.5155 | -4.84 | 6.59 |  |

**Suppl.Table 3:** V**alidation in liver with slope and intercept (i) of linear regression equation, and correlation coefficient (R2) as well as linearity given as back-calculated concentration (BCC), accuracy as relative error percentages (RE%) and as relative standard deviation (RSD) at given added concentrations.**

| Analyte | Parameter linear regression | | | | | | | Added concentration | | | | | | | | | | | | | | | |
| --- | --- | --- | --- | --- | --- | --- | --- | --- | --- | --- | --- | --- | --- | --- | --- | --- | --- | --- | --- | --- | --- | --- | --- |
|  | range(µM) 0.01-0.6 | | | | range(µM) 0.02-20 | | | 0.1µM (range 0.01-0.6) | | | | 1µM (range 0.02-20) | | | | 2µM (range 0.02-20) | | | | 10µM (range 0.02-20) | | | |
|  | slope | i | R2 | slope | | i | R2 | BCC | RE(%) | RSD(%) | BCC | | RE(%) | RSD(%) | BCC | | RE(%) | RSD(%) | BCC | | RE(%) | RSD(%) |  |
| dATP | 0.0635 | 0.0018 | 0.9996 | 0.0790 | | -0.0091 | 0.9993 | 0.1000 | -0.03 | 1.98 | 0.9154 | | -8.46 | 9.83 | 1.6870 | | -15.65 | 14.45 | 10.1358 | | 1.36 | 8.15 |  |
| Adenosine | 1.2144 | 0.2262 | 0.9995 | 1.2983 | | 0.2072 | 0.9993 | 0.0974 | -2.62 | 3.39 | 0.9685 | | -3.15 | 5.71 | 1.9945 | | -0.28 | 3.91 | 10.4198 | | 4.20 | 0.60 |  |
| ADP | 0.1106 | 0.0173 | 0.9827 | 0.2692 | | -0.0779 | 0.9963 | 0.0682 | -31.82 | 14.20 | 0.7659 | | -23.41 | 4.76 | 1.3582 | | -32.09 | 7.85 | 10.2546 | | 2.55 | 4.07 |  |
| AMP | 0.1830 | 0.2662 | 0.9514 | 0.2276 | | 0.2359 | 0.9988 | 0.0719 | -28.09 | 118.59 | 0.8952 | | -10.48 | 15.37 | 1.8690 | | -6.55 | 12.34 | 10.2933 | | 2.93 | 5.20 |  |
| ATP | 0.3067 | 0.0530 | 0.9955 | 0.2524 | | 0.0805 | 0.9996 | 0.0940 | -5.96 | 0.36 | 1.1490 | | 14.90 | 15.06 | 2.2679 | | 13.40 | 11.66 | 9.8632 | | -1.37 | 8.55 |  |
| cADPR | 0.1995 | -0.0001 | 0.9998 | 0.2384 | | -0.0346 | 0.9988 | 0.0963 | -3.71 | 2.86 | 0.9753 | | -2.47 | 2.67 | 1.8877 | | -5.62 | 3.79 | 9.9488 | | -0.51 | 3.59 |  |
| cAMP | 0.7108 | -0.0019 | 0.9998 | 0.8106 | | -0.1091 | 0.9988 | 0.0963 | -3.67 | 7.33 | 0.9918 | | -0.82 | 5.78 | 1.9131 | | -4.34 | 9.35 | 9.9177 | | -0.82 | 4.76 |  |
| cGMP | 0.3445 | -0.0011 | 0.9997 | 0.3872 | | -0.0532 | 0.9982 | 0.0988 | -1.21 | 6.95 | 1.0154 | | 1.54 | 7.69 | 1.9371 | | -3.15 | 10.06 | 10.0237 | | 0.24 | 7.75 |  |
| NAADP | 0.0283 | 0.0000 | 0.9998 | 0.0356 | | -0.0051 | 0.9992 | 0.0933 | -6.73 | 9.65 | 0.9365 | | -6.35 | 0.44 | 1.8329 | | -8.35 | 1.12 | 10.0340 | | 0.34 | 3.79 |  |
| NAD | 0.1520 | 0.0024 | 1.0000 | 0.1601 | | -0.0129 | 0.9989 | 0.0979 | -2.07 | 11.22 | 1.0458 | | 4.58 | 11.71 | 1.9834 | | -0.83 | 10.10 | 9.9156 | | -0.84 | 6.92 |  |
| NADH | 0.0652 | -0.0009 | 0.9973 | 0.1163 | | -0.0789 | 0.9550 | 0.0828 | -17.17 | 10.54 | 1.2038 | | 20.38 | 1.85 | 1.7878 | | -10.61 | 3.62 | 7.2928 | | -27.07 | 2.35 |  |
| NADP | 0.0378 | 0.0006 | 0.9987 | 0.0454 | | -0.0043 | 0.9993 | 0.0977 | -2.27 | 11.85 | 0.9710 | | -2.90 | 0.97 | 1.9133 | | -4.33 | 3.99 | 10.0803 | | 0.80 | 0.89 |  |
| NADPH | n.d. | n.d. | n.d.. | 0.0032 | | -0.0004 | 0.9993 | n.d. | n.d. | n.d. | 1.0640 | | 6.40 | 2.68 | 1.7971 | | -10.15 | 7.97 | 10.1979 | | 1.98 | 4.45 |  |
| Uridine | 0.5833 | 1.0974 | 0.9828 | 0.6302 | | 1.1016 | 0.9991 | 0.0937 | -6.25 | 118.37 | 0.9624 | | -3.76 | 22.48 | 2.1436 | | 7.18 | 14.47 | 10.4720 | | 4.72 | 3.13 |  |
| Hypoxanthine | 0.5851 | 2.0198 | 0.9230 | 0.6771 | | 1.9378 | 0.9988 | 0.0516 | -48.39 | 255.60 | 0.9600 | | -4.00 | 33.06 | 2.0357 | | 1.78 | 17.25 | 10.1233 | | 1.23 | 3.02 |  |
| Inosine | 1.1489 | 8.9525 | 0.5732 | 1.2512 | | 8.8937 | 0.9967 | -0.0881 | -188.08 | -630.48 | 0.8708 | | -12.92 | 64.69 | 2.0943 | | 4.72 | 37.27 | 10.9332 | | 9.33 | 7.31 |  |
| NAM | 0.1424 | 0.3558 | 0.9761 | 0.1485 | | 0.3511 | 0.9994 | 0.0820 | -18.02 | 155.82 | 1.0111 | | 1.11 | 4.65 | 2.0676 | | 3.38 | 3.08 | 10.2429 | | 2.43 | 3.94 |  |
| dNAD | 0.0352 | 0.0007 | 0.9967 | 0.0432 | | -0.0065 | 0.9985 | 0.1132 | 13.19 | 3.34 | 0.9990 | | -0.10 | 4.15 | 1.7867 | | -10.66 | 7.96 | 9.8969 | | -1.03 | 3.17 |  |
| Kynurenic acid | 0.5594 | -0.0015 | 0.9997 | 1.2450 | | -0.1727 | 0.9903 | 0.0976 | -2.41 | 12.07 | 0.5656 | | -43.44 | 6.79 | 1.0124 | | -49.38 | 10.68 | 11.1534 | | 11.53 | 16.88 |  |
| Kynurenine | 0.7328 | 0.0019 | 0.9999 | 0.7907 | | -0.0295 | 0.9993 | 0.0976 | -2.41 | 2.06 | 0.9604 | | -3.96 | 8.71 | 1.9411 | | -2.94 | 5.93 | 10.3958 | | 3.96 | 2.16 |  |
| NaADN | 0.0191 | -0.0001 | 0.9995 | 0.0202 | | -0.0021 | 0.9993 | 0.0923 | -7.75 | 10.57 | 0.9582 | | -4.18 | 3.72 | 1.9004 | | -4.98 | 0.91 | 10.0170 | | 0.17 | 2.34 |  |
| NAMN | 0.0857 | 0.0000 | 0.9994 | 0.0963 | | -0.0108 | 0.9990 | 0.0964 | -3.60 | 7.71 | 0.9920 | | -0.80 | 8.73 | 1.9050 | | -4.75 | 9.68 | 10.0671 | | 0.67 | 4.53 |  |
| NMN* | n.d. | n.d. | n.d.. | 0.0046 | | -0.0005 | 0.9988 | n.d. | n.d. | n.d. | 0.9976 | | -0.24 | 12.01 | 1.9214 | | -3.93 | 20.92 | 10.0471 | | 0.47 | 10.12 |  |
| Quinolinic acid# | 0.3153 | -0.0165 | 0.9995 | 0.3858 | | -0.1235 | 0.9953 | 0.1281 | 28.10 | 1.82 | 1.0994 | | 9.94 | 5.89 | 1.9129 | | -4.35 | 9.75 | 9.5071 | | -4.93 | 8.96 |  |
| Tryptophan | 0.5771 | 0.1232 | 0.9996 | 0.6513 | | 0.0703 | 0.9992 | 0.0961 | -3.89 | 19.77 | 0.9917 | | -0.83 | 7.70 | 1.9667 | | -1.67 | 8.82 | 10.1762 | | 1.76 | 4.03 |  |
| NR | 0.5446 | 0.0005 | 0.9999 | 0.6128 | | -0.0432 | 0.9992 | 0.0966 | -3.43 | 6.53 | 0.9747 | | -2.53 | 10.91 | 1.9051 | | -4.75 | 8.09 | 10.3238 | | 3.24 | 4.89 |  |

**Suppl.Table 4:** V**alidation in adipose tissue with slope and intercept (i) of linear regression equation, and correlation coefficient (R2) as well as linearity given as back-calculated concentration (BCC), accuracy as relative error percentages (RE%) and as relative standard deviation (RSD) at given added concentrations.**

| Analyte | Parameter linear regression | | | | | | | Added concentration | | | | | | | | | | | | |
| --- | --- | --- | --- | --- | --- | --- | --- | --- | --- | --- | --- | --- | --- | --- | --- | --- | --- | --- | --- | --- |
|  | range(µM) 0.01-0.6 | | | | range(µM) 0.02-20 | | | 0.1µM (range 0.01-0.6) | | | 1µM (range 0.02-20) | | | 2µM (range 0.02-20) | | | 10µM (range 0.02-20) | | | |
|  | slope | i | R2 | slope | | i | R2 | BCC | RE(%) | RSD(%) | BCC | RE(%) | RSD(%) | BCC | RE(%) | RSD(%) | BCC | RE(%) | RSD(%) |  |
| dATP | 0.0715 | 0.0016 | 0.9990 | 0.0799 | | -0.0044 | 0.9992 | 0.1067 | 6.66 | 2.54 | 0.9416 | -5.84 | 5.21 | 1.8939 | -5.30 | 3.30 | 10.3215 | 3.21 | 5.19 |  |
| Adenosine | 1.4998 | 0.0576 | 0.9998 | 1.3551 | | 0.2720 | 0.9968 | 0.0962 | -3.85 | 1.24 | 0.9482 | -5.18 | 4.84 | 2.1067 | 5.34 | 4.91 | 10.8586 | 8.59 | 5.40 |  |
| ADP | 0.1346 | 0.0202 | 0.9992 | 0.2309 | | -0.0275 | 0.9990 | 0.0939 | -6.09 | 1.55 | 0.8046 | -19.54 | 8.70 | 1.5961 | -20.20 | 0.97 | 10.1609 | 1.61 | 4.98 |  |
| AMP | 0.2414 | 0.1784 | 0.9878 | 0.2089 | | 0.1881 | 0.9997 | 0.0772 | -22.81 | 92.80 | 1.0827 | 8.27 | 4.45 | 2.0741 | 3.70 | 3.50 | 10.1839 | 1.84 | 2.96 |  |
| ATP | 0.2805 | 0.0258 | 0.9996 | 0.2582 | | 0.0277 | 0.9991 | 0.0929 | -7.06 | 8.50 | 1.0848 | 8.48 | 1.94 | 2.1708 | 8.54 | 0.46 | 10.1694 | 1.69 | 3.67 |  |
| cADPR | 0.2154 | -0.0003 | 0.9998 | 0.2320 | | -0.0152 | 0.9997 | 0.0953 | -4.68 | 1.00 | 0.9608 | -3.92 | 0.90 | 1.9462 | -2.69 | 3.38 | 10.1173 | 1.17 | 3.15 |  |
| cAMP | 0.7216 | -0.0019 | 0.9999 | 0.7525 | | -0.0255 | 0.9996 | 0.0977 | -2.35 | 3.59 | 0.9696 | -3.04 | 6.19 | 1.9841 | -0.79 | 7.49 | 10.2409 | 2.41 | 2.08 |  |
| cGMP | 0.3344 | -0.0004 | 0.9999 | 0.3550 | | -0.0188 | 0.9996 | 0.0998 | -0.20 | 0.01 | 0.9737 | -2.63 | 4.34 | 1.9735 | -1.33 | 4.62 | 10.1758 | 1.76 | 5.13 |  |
| NAADP | 0.0348 | -0.0001 | 0.9997 | 0.0367 | | -0.0034 | 0.9994 | 0.0957 | -4.27 | 5.12 | 0.9992 | -0.08 | 1.46 | 1.9537 | -2.32 | 3.69 | 9.8358 | -1.64 | 6.85 |  |
| NAD | 0.1457 | 0.0005 | 0.9997 | 0.1583 | | -0.0086 | 0.9993 | 0.0983 | -1.71 | 3.01 | 0.9613 | -3.87 | 2.99 | 1.9561 | -2.19 | 0.26 | 10.2890 | 2.89 | 8.86 |  |
| NADH | 0.0708 | -0.0012 | 0.9953 | 0.1291 | | -0.0831 | 0.9655 | 0.0759 | -24.06 | 9.34 | 1.1544 | 15.44 | 0.23 | 1.7048 | -14.76 | 0.24 | 7.9567 | -20.43 | 11.68 |  |
| NADP | 0.0422 | 0.0002 | 0.9998 | 0.0450 | | -0.0025 | 0.9996 | 0.0992 | -0.78 | 7.76 | 0.9916 | -0.84 | 3.98 | 1.9678 | -1.61 | 3.90 | 10.0732 | 0.73 | 6.22 |  |
| NADPH | n.d. | n.d. | n.d.. | 0.0027 | | 0.0007 | 0.9959 | n.d. | n.d. | n.d. | 0.9916 | -0.84 | 3.98 | 1.9678 | -1.61 | 3.90 | 10.0732 | 0.73 | 6.22 |  |
| Uridine | 0.7235 | 0.2071 | 0.9967 | 0.5983 | | 0.3078 | 0.9980 | 0.0870 | -12.98 | 29.60 | 0.9959 | -0.41 | 6.13 | 2.1403 | 7.01 | 9.58 | 10.6381 | 6.38 | 3.83 |  |
| Hypoxanthine | 0.7143 | 0.9957 | 0.9508 | 0.6555 | | 1.0299 | 0.9991 | 0.0233 | -76.68 | 530.53 | 0.9675 | -3.25 | 6.41 | 2.0952 | 4.76 | 9.82 | 10.4850 | 4.85 | 4.36 |  |
| Inosine | 1.6281 | 1.3473 | 0.9882 | 1.2799 | | 1.6107 | 0.9967 | 0.0690 | -31.03 | 112.93 | 0.9646 | -3.54 | 6.03 | 2.1726 | 8.63 | 11.53 | 10.8624 | 8.62 | 2.42 |  |
| NAM | 0.1575 | 0.0666 | 0.9915 | 0.1524 | | 0.0684 | 0.9997 | 0.0718 | -28.19 | 20.21 | 1.0110 | 1.10 | 2.54 | 2.0344 | 1.72 | 4.30 | 10.2640 | 2.64 | 6.20 |  |
| dNAD | 0.0423 | 0.0003 | 0.9991 | 0.0468 | | -0.0021 | 0.9997 | 0.1099 | 9.93 | 5.20 | 0.9687 | -3.13 | 4.81 | 1.9622 | -1.89 | 0.06 | 10.1778 | 1.78 | 7.61 |  |
| Kynurenic acid | 0.5448 | 0.0071 | 0.9998 | 1.1821 | | -0.3034 | 0.9965 | 0.0976 | -2.41 | 2.24 | 0.7192 | -28.08 | 5.82 | 1.1839 | -40.81 | 4.89 | 9.6879 | -3.12 | 0.02 |  |
| Kynurenine | 0.7950 | -0.0025 | 0.9998 | 0.7682 | | 0.0445 | 0.9987 | 0.0977 | -2.28 | 2.25 | 0.9491 | -5.09 | 7.01 | 2.0314 | 1.57 | 7.44 | 10.6138 | 6.14 | 3.37 |  |
| NaADN | 0.0189 | 0.0000 | 0.9998 | 0.0195 | | -0.0001 | 0.9992 | 0.0986 | -1.39 | 14.69 | 0.9917 | -0.83 | 7.72 | 2.0175 | 0.87 | 3.53 | 10.4366 | 4.37 | 3.57 |  |
| NAMN | 0.0853 | 0.0003 | 0.9999 | 0.0870 | | -0.0020 | 0.9996 | 0.1027 | 2.65 | 6.68 | 0.9978 | -0.22 | 3.58 | 2.0447 | 2.24 | 7.89 | 10.1740 | 1.74 | 3.32 |  |
| NMN* | 0.0041 | 0.0000 | 0.9974 | 0.0040 | | -0.0001 | 0.9998 | 0.1019 | 1.91 | 0.54 | 0.9628 | -3.72 | 9.68 | 2.0330 | 1.65 | 12.35 | 9.8430 | -1.57 | 5.04 |  |
| Quinolinic acid# | n.d. | n.d. | n.d.. | 0.3616 | | -0.0693 | 0.9987 | n.d. | n.d. | n.d. | 0.9896 | -1.04 | 3.80 | 1.9666 | -1.67 | 6.43 | 10.1550 | 1.55 | 7.87 |  |
| Tryptophan | 0.6077 | 0.0664 | 0.9993 | 0.6019 | | 0.0807 | 0.9992 | 0.0932 | -6.82 | 13.52 | 0.9662 | -3.38 | 3.22 | 2.0170 | 0.85 | 7.41 | 10.4742 | 4.74 | 2.22 |  |
| NR | 0.5291 | -0.0013 | 0.9996 | 0.5358 | | 0.0203 | 0.9986 | 0.0939 | -6.09 | 3.36 | 0.9400 | -6.00 | 8.79 | 1.9857 | -0.72 | 9.28 | 10.6588 | 6.59 | 0.29 |  |

**Suppl.Table 5:** V**alidation in plasma with slope and intercept (i) of linear regression equation, and correlation coefficient (R2) as well as linearity given as back-calculated concentration (BCC), accuracy as relative error percentages (RE%) and as relative standard deviation (RSD) at given added concentrations.**

| Analyte | Parameter linear regression | | | | | | Added concentration | | | | | | | | | | | |
| --- | --- | --- | --- | --- | --- | --- | --- | --- | --- | --- | --- | --- | --- | --- | --- | --- | --- | --- |
|  | range(µM) 0.01-0.6 | | | range(µM) 0.02-20 | | | 0.1µM (range 0.01-0.6) | | | 1µM (range 0.02-20) | | | 2µM (range 0.02-20) | | | 10µM (range 0.02-20) | | |
|  | slope | i | R2 | slope | i | R2 | BCC | RE(%) | RSD(%) | BCC | RE(%) | RSD(%) | BCC | RE(%) | RSD(%) | BCC | RE(%) | RSD(%) |
| dATP | 0.0704 | 0.0003 | 0.9998 | 0.0860 | -0.0214 | 0.9953 | 0.1065 | 6.51 | 7.89 | 1.0091 | 0.91 | 1.67 | 1.8155 | -9.22 | 1.56 | 9.2593 | -7.41 | 6.61 |
| Adenosine | 1.9721 | -0.0070 | 0.9995 | 1.7242 | 0.2388 | 0.9982 | 0.0961 | -3.89 | 8.92 | 0.9348 | -6.52 | 9.77 | 2.0928 | 4.64 | 4.60 | 10.5156 | 5.16 | 4.77 |
| ADP | 0.3292 | 0.0043 | 0.9996 | 0.3740 | -0.0558 | 0.9977 | 0.0967 | -3.32 | 7.18 | 1.0449 | 4.49 | 9.66 | 1.9321 | -3.40 | 12.72 | 9.4549 | -5.45 | 6.33 |
| AMP | 0.3798 | 0.0072 | 0.9994 | 0.3964 | -0.0251 | 0.9994 | 0.0992 | -0.83 | 18.12 | 1.0027 | 0.27 | 5.90 | 1.9669 | -1.65 | 13.42 | 9.8203 | -1.80 | 6.17 |
| ATP | 0.2346 | -0.0008 | 0.9992 | 0.3002 | -0.0925 | 0.9922 | 0.0958 | -4.18 | 8.82 | 1.0262 | 2.62 | 0.41 | 1.8295 | -8.52 | 1.58 | 8.9503 | -10.50 | 9.18 |
| cADPR | 0.3571 | -0.0005 | 0.9999 | 0.3992 | -0.0507 | 0.9989 | 0.0960 | -4.03 | 12.30 | 0.9898 | -1.02 | 4.58 | 1.9285 | -3.58 | 5.83 | 9.7798 | -2.20 | 0.91 |
| cAMP | 0.9336 | -0.0031 | 0.9997 | 0.9191 | -0.0221 | 0.9999 | 0.0971 | -2.86 | 17.14 | 0.9716 | -2.84 | 10.16 | 1.9871 | -0.65 | 13.70 | 10.0510 | 0.51 | 7.75 |
| cGMP | 0.4642 | -0.0018 | 0.9996 | 0.4616 | -0.0268 | 0.9997 | 0.0966 | -3.44 | 24.09 | 0.9829 | -1.71 | 14.07 | 1.9691 | -1.54 | 18.35 | 9.8538 | -1.46 | 14.81 |
| NAADP | 0.0258 | -0.0001 | 0.9987 | 0.0327 | -0.0087 | 0.9933 | 0.0989 | -1.15 | 8.81 | 1.0590 | 5.90 | 0.56 | 1.8990 | -5.05 | 8.08 | 8.9216 | -10.78 | 1.99 |
| NAD | 0.1612 | -0.0005 | 0.9998 | 0.1675 | -0.0138 | 0.9996 | 0.0963 | -3.74 | 0.21 | 0.9823 | -1.77 | 1.81 | 1.9207 | -3.97 | 7.49 | 9.7843 | -2.16 | 0.49 |
| NADH | 0.0880 | -0.0016 | 0.9944 | 0.0807 | -0.0043 | 0.9981 | 0.0778 | -22.20 | 14.85 | 1.1104 | 11.04 | 9.66 | 2.2759 | 13.80 | 12.75 | 9.5267 | -4.73 | 8.99 |
| NADP | 0.0509 | -0.0001 | 0.9998 | 0.0510 | -0.0027 | 0.9996 | 0.1013 | 1.32 | 9.51 | 0.9914 | -0.86 | 8.50 | 2.0158 | 0.79 | 5.29 | 9.7071 | -2.93 | 1.15 |
| NADPH | n.d. | n.d. | n.d.. | 0.0019 | -0.0003 | 0.9981 | n.d. | n.d. | n.d. | 1.1922 | 19.22 | 2.68 | 2.0398 | 1.99 | 11.89 | 9.4864 | -5.14 | 3.66 |
| Uridine | 0.7851 | 0.0974 | 0.9997 | 0.7536 | 0.1194 | 0.9999 | 0.0979 | -2.14 | 30.97 | 0.9625 | -3.75 | 13.73 | 2.0532 | 2.66 | 14.48 | 10.1201 | 1.20 | 10.86 |
| Hypoxanthine | 1.0746 | 0.0263 | 0.9995 | 1.0155 | 0.0811 | 0.9997 | 0.0969 | -3.12 | 16.10 | 0.9428 | -5.72 | 12.68 | 2.0371 | 1.85 | 11.97 | 10.2415 | 2.42 | 7.13 |
| Inosine | 2.1623 | -0.0077 | 0.9996 | 1.8186 | 0.3415 | 0.9972 | 0.0946 | -5.35 | 9.91 | 0.9369 | -6.31 | 8.00 | 2.1502 | 7.51 | 9.38 | 10.6004 | 6.00 | 5.85 |
| NAM | 0.1577 | 0.0003 | 0.9999 | 0.1556 | -0.0035 | 0.9999 | 0.0957 | -4.31 | 6.46 | 0.9820 | -1.80 | 2.53 | 2.0115 | 0.58 | 0.39 | 9.9303 | -0.70 | 0.39 |
| dNAD | 0.0464 | -0.0001 | 0.9996 | 0.0486 | -0.0037 | 0.9996 | 0.0954 | -4.59 | 1.74 | 1.0067 | 0.67 | 4.31 | 1.9149 | -4.26 | 6.96 | 9.9140 | -0.86 | 9.76 |
| Kynurenic acid | 1.5219 | -0.0161 | 0.9974 | 1.3366 | 0.2558 | 0.9939 | 0.0790 | -21.04 | 15.06 | 0.9660 | -3.40 | 42.09 | 2.0161 | 0.80 | 20.65 | 9.8044 | -1.96 | 13.81 |
| Kynurenine | 1.1230 | 0.0377 | 0.9993 | 1.0254 | 0.1260 | 0.9993 | 0.0960 | -3.99 | 18.81 | 0.9469 | -5.31 | 15.38 | 2.0505 | 2.53 | 10.10 | 10.3503 | 3.50 | 9.08 |
| NaADN | 0.0205 | 0.0001 | 0.9996 | 0.0222 | -0.0022 | 0.9991 | 0.1050 | 5.05 | 12.90 | 1.0321 | 3.21 | 0.83 | 1.9296 | -3.52 | 2.17 | 9.7310 | -2.69 | 2.15 |
| NAMN | 0.1440 | 0.0000 | 0.9996 | 0.1465 | -0.0102 | 0.9995 | 0.0968 | -3.19 | 10.62 | 1.0040 | 0.40 | 10.65 | 1.9680 | -1.60 | 15.24 | 9.7429 | -2.57 | 6.59 |
| NMN* | 0.0043 | 0.0000 | 0.9985 | 0.0044 | -0.0001 | 1.0000 | 0.1033 | 3.33 | 4.03 | 0.9822 | -1.78 | 3.62 | 1.9406 | -2.97 | 13.76 | 10.0217 | 0.22 | 5.93 |
| Quinolinic acid# | 0.8098 | 0.0107 | 0.9998 | 0.8569 | -0.0708 | 0.9994 | 0.0973 | -2.73 | 24.84 | 0.9832 | -1.68 | 14.19 | 1.9060 | -4.70 | 14.58 | 9.8414 | -1.59 | 6.44 |
| Tryptophan | 0.7283 | 1.7662 | 0.9668 | 0.7858 | 1.7592 | 0.9997 | 0.0716 | -28.36 | 456.87 | 0.8311 | -16.89 | 29.65 | 2.0214 | 1.07 | 24.58 | 10.1932 | 1.93 | 8.98 |
| NR | 0.5951 | -0.0014 | 0.9999 | 0.5104 | 0.0651 | 0.9987 | 0.0971 | -2.91 | 14.44 | 0.9327 | -6.73 | 14.14 | 2.1016 | 5.08 | 7.73 | 10.4298 | 4.30 | 4.36 |

**Suppl.Table 6:** V**alidation in cells with slope and intercept (i) of linear regression equation, and correlation coefficient (R2) as well as linearity given as back-calculated concentration (BCC), accuracy as relative error percentages (RE%) and as relative standard deviation (RSD) at given added concentrations.**

| Analyte | Parameter linear regression | | | | | | Added concentration | | | | | | | | | | | |
| --- | --- | --- | --- | --- | --- | --- | --- | --- | --- | --- | --- | --- | --- | --- | --- | --- | --- | --- |
|  | range(µM) 0.01-0.6 | | | range(µM) 0.02-20 | | | 0.1µM (range 0.01-0.6) | | | 1µM (range 0.02-20) | | | 2µM (range 0.02-20) | | | 10µM (range 0.02-20) | | |
|  | slope | i | R2 | slope | i | R2 | BCC | RE(%) | RSD(%) | BCC | RE(%) | RSD(%) | BCC | RE(%) | RSD(%) | BCC | RE(%) | RSD(%) |
| dATP | 0.0303 | 0.0045 | 0.9989 | 0.0857 | -0.0200 | 0.9943 | 0.1069 | 6.91 | 20.91 | 0.7282 | -27.18 | 6.51 | 1.7797 | -11.01 | 28.86 | 10.4056 | 4.06 | 36.99 |
| Adenosine | 1.2808 | 0.0073 | 0.9991 | 1.2820 | 0.1547 | 0.9939 | 0.0972 | -2.77 | 6.39 | 0.8608 | -13.92 | 16.71 | 1.9583 | -2.09 | 3.82 | 11.1882 | 11.88 | 15.91 |
| ADP | 0.0492 | 0.0902 | 0.5194 | 0.2350 | 0.0209 | 0.9926 | -0.0349 | -134.85 | -828.61 | 0.9486 | -5.14 | 15.78 | 2.7448 | 37.24 | 45.78 | 10.7727 | 7.73 | 47.19 |
| AMP | 0.1173 | 0.1197 | 0.7526 | 0.2882 | 0.0850 | 0.9911 | -0.0126 | -112.61 | -1056.27 | 0.9198 | -8.02 | 30.57 | 2.7961 | 39.81 | 3.88 | 11.2614 | 12.61 | 16.85 |
| ATP | 0.0062 | 0.0987 | 0.0145 | 0.1028 | 0.0630 | 0.9919 | -1.2101 | -1310.08 | -148.85 | -0.7258 | -172.58 | -25.08 | 9.1821 | 359.11 | 73.48 | 10.7291 | 7.29 | 41.30 |
| cADPR | 0.1443 | 0.0010 | 0.9994 | 0.3225 | -0.0246 | 0.9860 | 0.1035 | 3.50 | 9.12 | 0.8896 | -11.04 | 13.79 | 2.0033 | 0.16 | 3.68 | 11.1505 | 11.51 | 16.81 |
| cAMP | 0.4898 | 0.0003 | 0.9983 | 0.7400 | -0.0622 | 0.9957 | 0.0979 | -2.11 | 5.01 | 0.9040 | -9.60 | 16.29 | 1.9948 | -0.26 | 1.08 | 10.8421 | 8.42 | 12.68 |
| cGMP | 0.1142 | -0.0005 | 0.9981 | 0.1748 | -0.0171 | 0.9956 | 0.1001 | 0.11 | 5.35 | 0.8552 | -14.48 | 16.80 | 1.9101 | -4.49 | 4.91 | 10.7354 | 7.35 | 16.23 |
| NAADP | 0.0091 | 0.0003 | 0.9982 | 0.0273 | -0.0038 | 0.9848 | 0.1017 | 1.73 | 9.94 | 0.9518 | -4.82 | 23.85 | 1.9090 | -4.55 | 12.77 | 11.3772 | 13.77 | 36.02 |
| NAD | 0.0467 | 0.0021 | 0.9983 | 0.1149 | -0.0094 | 0.9859 | 0.1102 | 10.15 | 12.89 | 0.9320 | -6.80 | 23.48 | 2.1041 | 5.20 | 2.97 | 11.3408 | 13.41 | 11.22 |
| NADH | 0.0811 | -0.0060 | 0.9874 | 0.1227 | -0.0202 | 0.9970 | 0.0819 | -18.10 | 3.87 | 0.8381 | -16.19 | 19.72 | 2.0616 | 3.08 | 0.88 | 10.6652 | 6.65 | 21.42 |
| NADP | 0.0112 | 0.0005 | 0.9989 | 0.0318 | -0.0036 | 0.9817 | 0.1072 | 7.16 | 6.22 | 0.8679 | -13.21 | 7.90 | 2.0300 | 1.50 | 10.11 | 11.8357 | 18.36 | 26.17 |
| NADPH | 0.0022 | -0.0010 | 0.9867 | 0.0017 | 0.0002 | 0.9741 | n.d. | n.d. | n.d. | 0.8945 | -10.55 | 16.82 | 1.2651 | -36.75 | 7.38 | 11.6992 | 16.99 | 6.85 |
| Uridine | 0.3543 | 0.0022 | 0.9987 | 0.4202 | -0.0207 | 0.9985 | 0.0985 | -1.54 | 2.49 | 0.8567 | -14.33 | 14.68 | 1.9506 | -2.47 | 4.69 | 10.5817 | 5.82 | 20.97 |
| Hypoxanthine | 0.4728 | 0.0343 | 0.9992 | 0.5597 | -0.0129 | 0.9991 | 0.0955 | -4.49 | 19.18 | 0.8474 | -15.26 | 17.71 | 1.9544 | -2.28 | 8.12 | 10.4414 | 4.41 | 15.05 |
| Inosine | 0.9255 | 0.0196 | 0.9996 | 0.8611 | 0.2991 | 0.9804 | 0.0989 | -1.13 | 19.00 | 0.8937 | -10.63 | 12.12 | 2.0245 | 1.22 | 10.16 | 11.4492 | 14.49 | 26.50 |
| NAM | 0.1364 | 0.0068 | 0.9992 | 0.1546 | -0.0159 | 0.9985 | 0.0906 | -9.39 | 13.53 | 0.8617 | -13.83 | 20.95 | 2.0127 | 0.63 | 3.19 | 9.9951 | -0.05 | 10.11 |
| dNAD | 0.0519 | 0.0002 | 0.9994 | 0.1029 | -0.0077 | 0.9879 | 0.1017 | 1.66 | 6.00 | 0.9103 | -8.97 | 21.36 | 1.9814 | -0.93 | 3.36 | 11.3469 | 13.47 | 14.93 |
| Kynurenic acid | 2.3513 | -0.0505 | 0.9819 | 1.6650 | 0.5940 | 0.9865 | 0.0544 | -45.65 | 3.86 | 0.8552 | -14.48 | 16.77 | 1.7557 | -12.21 | 8.44 | 10.7500 | 7.50 | 13.76 |
| Kynurenine | 0.3531 | -0.0034 | 0.9978 | 0.4795 | -0.0744 | 0.9982 | 0.0908 | -9.15 | 8.63 | 0.8552 | -14.48 | 17.29 | 1.9396 | -3.02 | 9.56 | 10.5626 | 5.63 | 15.01 |
| NaADN | 0.0078 | 0.0000 | 0.9987 | 0.0171 | -0.0006 | 0.9824 | 0.1055 | 5.48 | 1.17 | 0.9328 | -6.72 | 25.65 | 2.0890 | 4.45 | 4.04 | 11.5438 | 15.44 | 9.53 |
| NAMN | 0.0011 | -0.0004 | 0.9951 | 0.0010 | -0.0002 | 0.9950 | n.d. | n.d. | n.d. | 0.9225 | -7.75 | 10.60 | 1.5604 | -21.98 | 9.05 | 10.7230 | 7.23 | 4.36 |
| NMN* | 0.2839 | -0.0004 | 0.9995 | 0.5218 | -0.1174 | 0.9981 | 0.0969 | -3.07 | 5.63 | 0.8649 | -13.51 | 17.91 | 2.0701 | 3.50 | 0.17 | 10.1951 | 1.95 | 10.07 |
| Quinolinic acid# | 0.4597 | 0.0045 | 0.9986 | 0.5680 | -0.0197 | 0.9971 | 0.0919 | -8.07 | 11.78 | 0.8795 | -12.05 | 14.94 | 1.9664 | -1.68 | 10.14 | 10.8286 | 8.29 | 16.98 |
| Tryptophan | 0.0677 | 0.0006 | 0.9855 | 0.1280 | -0.0318 | 0.9984 | 0.0749 | -25.07 | 14.20 | 0.9501 | -4.99 | 10.47 | 2.3318 | 16.59 | 11.08 | 10.1679 | 1.68 | 22.56 |
| NR | 0.1554 | 0.0005 | 0.9994 | 0.1641 | -0.0235 | 0.9978 | 0.0985 | -1.47 | 18.54 | 0.8193 | -18.07 | 5.27 | 1.9473 | -2.63 | 5.28 | 9.9509 | -0.49 | 0.40 |
